# Isolation, characterisation and description of the roseoflavin producer *Streptomyces berlinensis* sp. nov.

**DOI:** 10.1111/1758-2229.13266

**Published:** 2024-04-23

**Authors:** Jimmy Jonathan Liunardo, Sebastien Messerli, Ann‐Kathrin Gregotsch, Sonja Lang, Kerstin Schlosser, Christian Rückert‐Reed, Tobias Busche, Jörn Kalinowski, Martin Zischka, Philipp Weller, Imen Nouioui, Meina Neumann‐Schaal, Chandra Risdian, Joachim Wink, Matthias Mack

**Affiliations:** ^1^ Institute for Technical Microbiology, Department of Biotechnology Mannheim University of Applied Sciences Mannheim Germany; ^2^ Medical School East Westphalia‐Lippe Bielefeld University Bielefeld Germany; ^3^ Technology Platform Genomics, Center for Biotechnology Bielefeld University Bielefeld Germany; ^4^ Institute for Instrumental Analytics and Bioanalytics, Department of Biotechnology Mannheim University of Applied Sciences Mannheim Germany; ^5^ Leibniz‐Institute DSMZ‐German Collection of Microorganisms and Cell Cultures Braunschweig Germany; ^6^ Department of Microbial Strain Collection Helmholtz Centre for Infection Research Braunschweig Germany; ^7^ Research Center for Applied Microbiology National Research and Innovation Agency (BRIN) Bandung Indonesia; ^8^ German Centre for Infection Research (DZIF) Partner Site Hannover‐Braunschweig Braunschweig Germany

## Abstract

The Gram‐positive bacteria *Streptomyces davaonensis* and *Streptomyces cinnabarinus* have been the only organisms known to produce roseoflavin, a riboflavin (vitamin B_2_) derived red antibiotic. Using a selective growth medium and a phenotypic screening, we were able to isolate a novel roseoflavin producer from a German soil sample. The isolation procedure was repeated twice, that is, the same strain could be isolated from the same location in Berlin 6 months and 12 months after its first isolation. Whole genome sequencing of the novel roseoflavin producer revealed an unusual chromosomal arrangement and the deposited genome sequence of the new isolate (G + C content of 71.47%) contains 897 genes per inverted terminal repeat, 6190 genes in the core and 107 genes located on an illegitimate terminal end. We identified the roseoflavin biosynthetic genes *rosA*, *rosB* and *rosC* and an unusually high number of riboflavin biosynthetic genes. Overexpression of *rosA*, *rosB* and *rosC* in *Escherichia coli* and enzyme assays confirmed their predicted functions in roseoflavin biosynthesis. A full taxonomic analysis revealed that the isolate represents a previously unknown *Streptomyces* species and we propose the name *Streptomyces berlinensis* sp. nov. for this roseoflavin producer.

## Graphical abstract

Two roseoflavin producing species have been isolated from Asian soil samples. We anticipated that synthesis of this antibiotic and vitamin analogue would be more widespread and isolated a novel roseoflavin releasing bacterium, *Streptomyces berlinensis*, from a Berlin (Germany) soil sample.

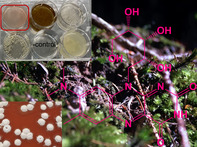

## INTRODUCTION

**FIGURE 1:**
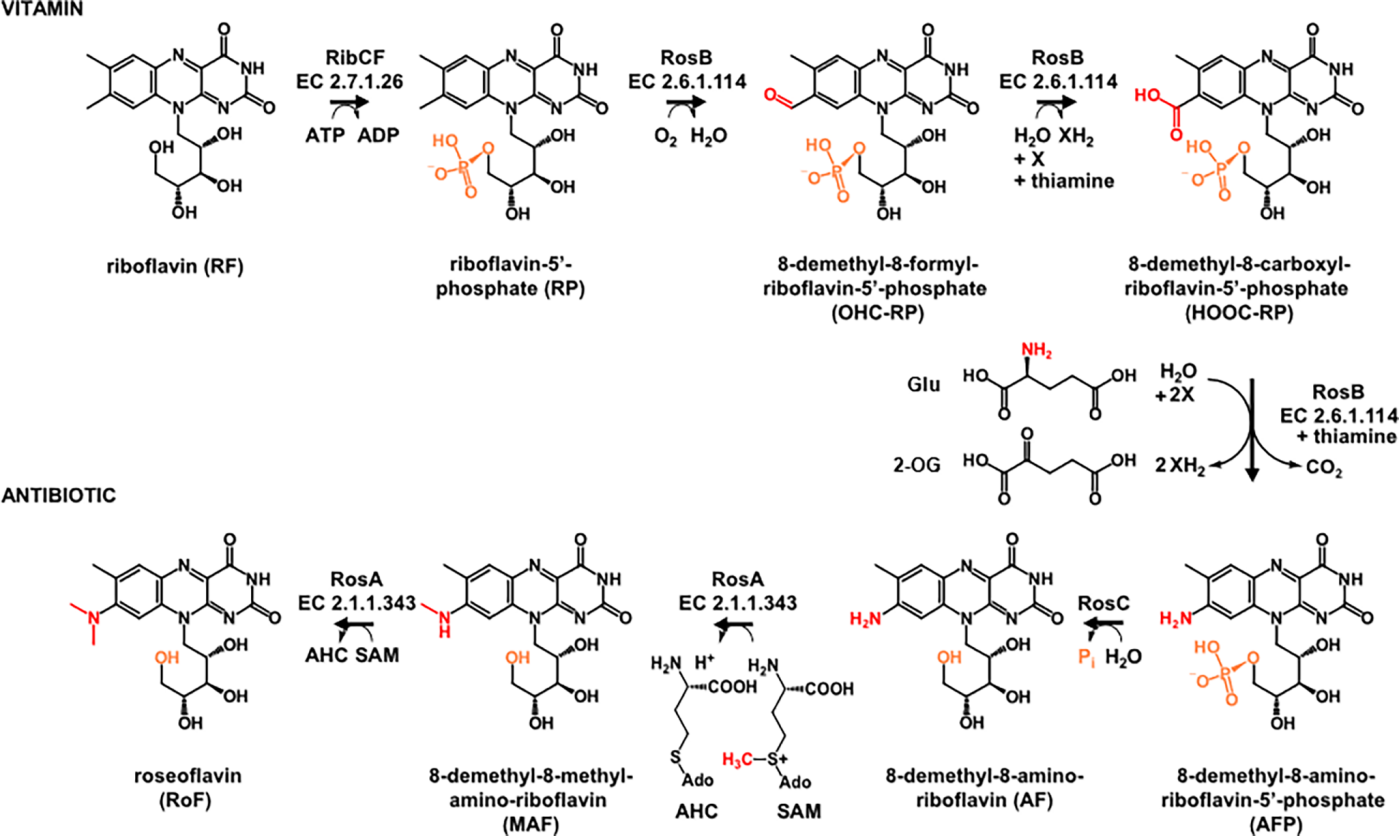
Roseoflavin biosynthesis. The path from riboflavin (vitamin B_2_) to the antibiotic roseoflavin is shown (Jhulki et al., 2016; Konjik et al., 2017; Schneider et al., 2020; Schwarz et al., 2016). An oxidative cascade replaces the C8 methyl of riboflavin‐5′‐phosphate (RP or FMN) with an amino group. The amino group donor is glutamate (Glu) causing release of 2‐oxoglutarate (2‐OG). The methyl donor of the final reaction steps is *S*‐adenosyl methionine (SAM).

Streptomycetes are high G + C Gram‐positive, spore‐forming bacteria of the family Streptomycetaceae (order Kitasatosporales), and the corresponding genus *Streptomyces* includes more than 500 species. Streptomycetes are widely distributed in soils and exceed in abundance the other soil bacterial genera (Kämpfer et al., 2014). *Streptomyces davaonensis* (DSM 101723^T^ = JCM 4913^T^) was first isolated from a Philippine soil sample within the framework of a screening programme for novel antibiotics (Otani et al., 1974). In this first description (Otani et al., 1974) the isolate tentatively was named ‘*S. davawensis*’ but the bacterium was renamed ‘*S. davaonensis*’ as a result of a more recent work in which this organism was described as a valid species (Landwehr et al., 2018). *S. davaonensis* releases a red compound (8‐demethyl‐8‐(dimethylamino)riboflavin), which exhibits antibiotic activity against a variety of Gram‐positive and Gram‐negative bacteria. Due to its red colour and its structural similarity to riboflavin the antibiotic was named roseoflavin (Otani et al., 1974). Roseoflavin and its precursor 8‐demethyl‐8‐aminoriboflavin (AF) are the only known natural riboflavin analogues with antibiotic properties (Pedrolli et al., 2013). Gram‐positive bacteria appear to represent the main target organisms for roseoflavin as riboflavin transporters, which also import the structurally very similar roseoflavin, are especially widespread in this group (Gutierrez‐Preciado et al., 2015; Hemberger et al., 2011; Vogl et al., 2007). Following uptake, the ‘prodrug’ roseoflavin is ‘activated’ to roseoflavin‐5′‐phosphate (or roseoflavin mononucleotide, RoFMN) and roseoflavin adenine dinucleotide (RoFAD) by promiscuous flavokinases (EC 2.7.1.26) and flavin adenine dinucleotide (FAD) synthetases (EC 2.7.7.2) (Langer et al., 2013). RoFMN and RoFAD have different physicochemical properties when compared to the cofactors flavin mononucleotide (FMN) and FAD and have the potential to reduce the activity of some (if not all) flavoproteins present within a target cell (Langer et al., 2013). In addition, FMN riboswitches, which regulate riboflavin biosynthesis and transport, were found to be turned off upon binding RoFMN resulting in strongly reduced riboflavin levels and reduced growth (Lee et al., 2009; Ott et al., 2009; Wang et al., 2017). We refer to such FMN riboswitches as ‘RoFMN‐sensitive FMN riboswitches’. Roseoflavin resistance of the roseoflavin producer *S. davaonensis* is conferred by a specialised FMN riboswitch, which is not negatively affected by RoFMN and thus represents a ‘RoFMN‐insensitive FMN riboswitch’ (Pedrolli et al., 2012). A yet unknown roseoflavin exporter most likely contributes to roseoflavin resistance (Schneider & Mack, 2021). Only three enzymes, RosA, RosB and RosC, are necessary to convert riboflavin‐derived FMN into the potent antibiotic roseoflavin (Schneider et al., 2020; Schwarz et al., 2016) (Figure 1). Unlike many other secondary metabolite genes, the *ros*‐genes are expressed from two independent gene clusters rather than from a single one (Kißling et al., 2020). The *rosA* gene cluster consists of 10 genes (including *rosA* and *rosC*), whereby the function of the remaining eight genes is not known. The *rosB* cluster is strongly expressed during the roseoflavin production phase and comprises the genes *rosG*, *ribE2*, *ribX*, *ribY* and *rosB*. The gene *rosG* encodes a putative γ‐glutamyltransferase/glutathione hydrolase‐like enzyme. Possibly, this enzyme generates glutamate, which is the amino group donor in the reaction catalysed by RosB (Figure 1 **)** (Konjik et al., 2017). The gene *ribE2* encodes a riboflavin synthase which increases levels of the roseoflavin precursor riboflavin (Kißling et al., 2020). The genes *ribXY* encode a highly active riboflavin import system and contribute to enhanced cytoplasmic riboflavin levels as well. When we compared the 16S rRNA gene sequence of *S. davaonensis* with reference 16S rRNA gene sequences, *Streptomyces cinnabarinus* was the closest relative and we found that this bacterium was a roseoflavin producer as well (Jankowitsch et al., 2012).

The aim of the present work was to isolate a novel roseoflavin producer. A new bacterial species that synthesises roseoflavin and contains the corresponding *ros* genes could indeed be isolated from a Berlin soil sample, and we named this bacterium *Streptomyces berlinensis*.

## EXPERIMENTAL PROCEDURES

### Chemicals

Roseoflavin was purchased from Chemos (Regenstauf, Germany). Thiamine, l‐glutamic acid, FMN and *S*‐adenosyl methionine (SAM) were purchased from AppliChem GmbH (Darmstadt, Germany). 8‐Demethyl‐8‐aminoriboflavin (AF) was a gift from Peter Macheroux (Technical University of Graz, Austria) and the corresponding 5′‐phosphate was prepared enzymatically from AF as described (Pedrolli & Mack, 2014).

### Bacterial strains and growth conditions

Activated charcoal (Carl Roth GmbH + Co. KG, Karlsruhe, Germany, product X865.1) was used to prepare CV agar which was used to enrich Streptomycetes (1.5 g charcoal/L; 17 g/L agar‐agar) (You & Park, 1996). To 1 L of CV 40 mg/L of nystatin (in dimethyl sulfoxide, DMSO) were added as an antifungal agent. Moreover, the following compounds were added (0.5 mg/L): Thiamine‐HCI, riboflavin (in DMSO), 4‐aminobenzoic acid, niacin, inositol, calcium‐pantothenate and pyridoxin‐HCI. Biotin was added at 0.25 mg/L. The pH of CV agar was adjusted to 7.2 with 1 M NaOH or 1 N HCl before autoclaving. Vitamins and nystatin were sterilised by filtration and added to the autoclaved media at 45°C. Soil samples (10 g) were added to a sterile Erlenmeyer flask (100 mL) and suspended in 50 mL 0.9% (w/V) Na‐polyphosphate (Na(PO_3_)_n_, Merck, Darmstadt, Germany, product 1.06529.1000) prepared with water. A stirring bar (1 cm) was added and the suspension was stirred at room temperature for at least 30 min. Dilutions of these suspensions were generated (1:100 and 1:1000) and 100 μL of these suspensions were spread on CV‐agar and incubated aerobically at 30°C for 7–14 days. The resulting colonies were transferred to maltose soybean (MS) agar, containing maltose (20 g/L), soybean meal (20 g/L) and agar‐agar (20 g/L). Spores from isolated colonies from MS agar were transferred to a nutrient broth (YS) in a 6‐well cell culture plate. YS contained yeast extract (2 g/L) and soluble potato starch (10 g/L) (pH 7.2). The 6‐well plates were incubated aerobically at 30°C and 130 rpm for 10 days while kept in a plastic box with damp paper towels. *S. berlinensis* was routinely grown on YS and MS whereby only YS supported roseoflavin production. ISP media were used for characterising *Streptomyces* species according to the International *Streptomyces* Project (ISP) (Shirling & Gottlieb, 1966).

### Analysis of flavins

The analysis of flavins was carried out using high‐performance liquid chromatography coupled to a diode array and fluorescence detector (HPLC‐DAD/FLD). To analyse flavin levels in culture supernatants of Streptomycetes, the cells were separated from the culture broth by centrifugation (6000× *g*, 10 min). Starch‐containing samples (derived from cultures grown in YS) were treated with α‐amylase for 10 min at room temperature prior to treatment with 5% (w/v) trichloroacetic acid (TCA) for 10 min at 4°C. Subsequently, samples were centrifuged (8000× *g*, 10 min, 4°C) and filtered (0.2 μm regenerated cellulose membrane filter) prior to HPLC analysis. HPLC analysis of roseoflavin was performed at a flow rate of 0.2 mL per min at 50°C using a Phenomenex Biphenyl HPLC column (2.6 μm particle size, 150 mm × 2.1 mm; Aschaffenburg, Germany) in combination with the Agilent 1260 Infinity system. Roseoflavin was separated using a gradient elution programme. Starting at 15% (v/v) methanol and 85% (v/v) buffer A (10 mM formic acid, 10 mM ammonium formate; pH 3.7), the methanol concentration was increased to 100%. A roseoflavin standard stock solution was used as references. HPLC‐MS/MS analysis of roseoflavin was performed in ESI positive mode on a Micromass Ultima triple quadrupole mass spectrometer, coupled to a Waters 2695 HPLC system, using a Kinetex 5 μm C18 100 Å, 100 × 4 mm column (Phenomenex). The gradient elution at a flow rate of 1.0 mL/min with solvents A (0.1% v/v formic/10 mM ammonium formate w/v in H_2_O) and B (0.1% v/v formic acid in methanol) started at 85% A to increase to 100% B after 10 min, to hold for additional 5 min. Samples were dissolved in a 50:50 v/v mixture of A and B and were kept at 10°C in the autosampler until measurement. The column was kept at 20°C, injection volume was 10 μL. The MRM method was generated in ESI positive mode based on the protonated precursor ion [M + H] + with m/z 406 and two transitions, m/z 406‐> 272 as quantifier ion and m/z 406‐> 174 as qualifier ion. Collision energy was at 16 V and 22 V, respectively. ESI capillary voltage was set to 3 kV, cone voltage was 40 V, desolvatisation temperature was at 350°C, source temperature was kept at 120°C. Desolvatisation gas flow was at 650 L/h. All analysis and data processing was done with MassLynx V4.1 (Waters Corporation).

### *Heterologous expression of rosA, rosB and rosC in* E. coli

The *S. berlinensis ros* genes were overexpressed in *E. coli* Rosetta 2 (DE3) (Merck KGaA, Darmstadt, Germany) using the expression plasmid pET24a(+). Three primer pairs (Table S9) introduced restriction endonuclease sites (*Nde*I and *Hin*dIII) for cloning. *E. coli* Rosetta 2 (DE3) was transformed using a standard protocol for transformation of chemically competent *E. coli* cells (Sambrook et al., 1989). For expression, the resulting strains were aerobically grown at 30°C in 100 mL LB (10 g/L NaCl, 10 g/L tryptone and 5 g/L yeast extract). Ampicillin (100 mg/L) was added when necessary. Expression was induced by adding 0.4 mM isopropylthiogalactopyranoside (IPTG) at an optical density (OD_600_) of 0.6. After 5 h of further aerobic incubation, cells were harvested by centrifugation (8000 *g*, 15 min, 4°C). The cell pellets were washed twice with sterile water and stored at −20°C until cell disruption.

### Cell‐disruption and enzyme assays

Frozen cell pastes of *E. coli* Rosetta 2 (DE3) overproducing RosA, RosB and RosC were lysed using 0.1 mm glass beads and a standard protocol with the FastPrep‐24TM 5G instrument from MP Biomedicals Germany (Eschwege, Germany). Cell debris and unbroken cells were removed by centrifugation (6000 g, 4°C, 10 min). The cleared lysate was directly used for testing enzyme activity. Protein was estimated by the method of Bradford. RosA activity was measured in a final volume of 1 mL of 50 mM Tris–HCl (pH 8.0) containing 1 mg total protein derived from a cell‐free extract, 200 μM AF and 2 mM SAM (Jankowitsch et al., 2011). The mixture was first incubated at 37°C for 5 min, and the reaction subsequently was started by addition of SAM. RosB activity was measured at 39°C in a final volume of 1 mL of 100 mM bis‐tris‐propane (BTP) (pH 8.0) containing 233 μM riboflavin‐5′‐phosphate, 20 μM CaCl2, 10 mM thiamine, 5 mM NAD^+^ and 5 mM L‐glutamic acid (5 mg total protein from a cell‐free extract was used) (Konjik et al., 2017). RosC activity was measured at 37 in 100 mM BTP containing 100 μM AFP and 20 μM CaCl_2_ (pH 7.6) (0.5 mg total protein from a cell‐free extract was used) (Schneider et al., 2020). Mixtures were equilibrated at 37°C for 5 min and reactions were started by adding cell‐free extracts. For determination of flavin levels, aliquots were removed and treated with 5% (w/v) TCA. Samples were centrifuged (8000 *g*, 10 min, 4°C) and filtered (0.2 μm regenerated cellulose membrane filter) prior to analysis by HPLC‐DAD.

### Isolation of genomic DNA

Preparation of *S. berlinensis* genomic DNA was performed using the NucleoSpin Microbial DNA Kit from Macherey‐Nagel (Düren, Germany). Cells were lysed two times for 20 sec at 4 ms^−1^ using a FastPrep‐24TM 5G instrument from MP Biomedicals Germany (Eschwege, Germany) and MN Bead Tubes Type C from Macherey‐Nagel (Düren, Germany).

### Genome sequencing and assembly

Long and short DNA reads were generated by Nanopore and Illumina sequencing, respectively. For library preparation, the TruSeq DNA PCR‐free high‐throughput library prep kit (Illumina) and the SQK‐LSK112 sequencing kit (Oxford Nanopore Technologies [ONT]) were used without prior shearing of the DNA. To generate the short reads, a 2 × 300‐nucleotide run (MiSeq reagent kit v3, 600 cycles) was executed. The long reads were generated on a GridION platform using an R10.4 flow cell. Base calling and demultiplexing were performed using guppy v6.1.5 with the super‐accurate base calling model. Assemblies were done using flye v.2.9 (Kolmogorov et al., 2019) for the Nanopore long read data and newbler v2.8 (Miller et al., 2010) for the Illumina short read data. After polishing of the flye‐based assembly using medaka v1.5.0 and pilon v1.22 (Walker et al., 2014) using bowtie2 (Langmead & Salzberg, 2012) for mapping, the respective flye and newbler assemblies were combined in consed v28.0 (Gordon et al., 1998). The resulting contigs representing the genome parts were annotated using the PGAP pipeline (Li et al., 2021; Tatusova et al., 2016) and are available at GenBank under accession numbers CP115393 and OQ979106.

### Prediction of open reading frames and functional annotation

Potential protein‐coding sequences (CDSs) were identified using the GenDB genome annotation tool (Meyer et al., 2003). For the identification of CDSs, the prokaryotic gene finders Prodigal (Hyatt et al., 2010) and GISMO (Krause et al., 2007) were employed. To improve results and to allow for easier manual correction, further methods were applied by means of the Reganor software (Linke et al., 2006) utilising the gene prediction tools Glimmer (Delcher et al., 1999) and CRITICA (Badger & Olsen, 1999). To link the identified open reading frames to potential functions different software packages were used to analyse DNA and amino acid sequences. Similarity‐based searches against public and/or proprietary nucleotide‐ and protein‐databases were performed employing BLASTp (Coulson, 1994) and RPS‐BLAST (Yang et al., 2020). From significant sequence similarities of the major section of a gene similar functions in *S. berlinensis* were concluded. Enzymatic classification was carried out on the basis of enzyme commission (EC) numbers (Bairoch, 2000). Primarily, they were derived from the PRIAM database using the PRIAM search tool. As a second approach, when no PRIAM result was available, EC‐number annotations were derived from searches against the Kyoto Encyclopedia of Genes and Genomes databases (Kanehisa & Goto, 2000). Further functional gene annotations were performed using the database of the cluster of orthologous groups of proteins (COG) classification system (Tatusov et al., 2003). Secondary metabolite gene cluster were identified using the antiSMASH software pipeline, which allows automated identification of gene clusters of all known secondary metabolite compound classes (Medema et al., 2011). To identify potential transmembrane proteins, the software TMHMM (Krogh et al., 2001) was used. Finally, the genome was examined manually using the BLASTp programme (Coulson, 1994). Computational identification of riboswitches in *S. berlinensis* was done by performing profile searches. For this purpose the covariance models (CM) of known riboswitch families were downloaded from the Rfam database (version 10.0) (Burge et al., 2013) and ‘cmsearch’, which is part of ‘infernal 1.0.2’ (Burge et al., 2013) was used to search the genome of *S. berlinensis* for sequences that match the CMs. Matches that exceeded the gathering threshold of the CMs were reported as family members.

### Phylogenetic analysis

The 16S rRNA gene sequence of strain 14.2 (1528 bp) was obtained from the genome sequence of strain 14.2 and was used for sequence comparisons using the EZBioCloud server (www.ezbiocloud.net; accessed on 8 April 2023) (Lee et al., 2017). Phylogenetic relations between strain 14.2 and closely related strains were inferred by the GGDC (Genome‐to‐Genome Distance Calculator) web server (http://ggdc.dsmz.de; accessed on 8 April 2023) (Meier‐Kolthoff et al., 2022) using the DSMZ phylogenomics pipeline (Meier‐Kolthoff et al., 2014). A multiple sequence alignment was conducted using MUSCLE (Edgar, 2004). The maximum likelihood (ML) and maximum parsimony (MP) trees were inferred using RAxML (Stamatakis, 2014) and tree analysis using new technology (TNT) (Goloboff et al., 2008), respectively.

### *Comparative analysis of* Streptomyces *genomes*

The genome sequence of strain 14.2 was compared with the genome sequence of closely related type strains. The orthologous average nucleotide identity (orthoANI) (Lee et al., 2016) and digital DNA–DNA hybridisation (dDDH) (Meier‐Kolthoff et al., 2013) values were determined using the ANI calculator tool from the EZBioCloud platform (www.ezbiocloud.net; accessed on 8 April 2023) and Type (Strain) Genome Server (https://tygs.dsmz.de; accessed on 8 April 2023), respectively.

### Morphological and physiological characterisation

With regard to the morphological description of colonies and analysis of melanin production strain 14.2 was precultured in liquid ISP2 and after 5 days plated on ISP2, ISP3, ISP4, ISP5, ISP6 and ISP7 agar (Shirling & Gottlieb, 1966). An aliquot (1 mL) of the liquid cultures was plated on ISP media and incubated for 10 days at 28°C. The plates were visually inspected with regard to growth, the colour of the substrate mycelium, aerial mycelium formation and production of water‐soluble pigments. Melanin formation was monitored on ISP6 and ISP7 plates. Utilisation of carbohydrates was monitored using the method of Shirling and Gottlieb (Shirling & Gottlieb, 1966). Temperature optimum and pH tolerance were analysed on ISP2. Physiological fingerprints were carried out employing API ZYM™ strips according to the instructions of the supplier (bioMerieux, Nürtingen, Germany).

### Chemotaxonomic analyses

Strain 14.2, DSM 40467^T^ and DSM 101723^T^ were grown aerobically (200 rpm) for 3 days at 28°C employing the liquid media DSMZ 535 (strains 14.2^T^ and DSM 101723^T^) and DSMZ 65 (strain DSM 40467^T^). The cells were harvested by centrifugation, washed three times with sterile distilled water and freeze‐dried. The diaminopimelic acid contents of the cell walls, their whole cell sugar contents and their polar lipid profiles were determined as described in a previous work (Landwehr et al., 2018). The menaquinone pattern of the strains was identified following a standard protocol (Alderson et al., 1985). Extraction of fatty acids was performed following a standard protocol as well as gas chromatographic analysis carried out on an Agilent instrument (model 6890 N) (Landwehr et al., 2018). The peaks were identified by the Standard Microbial Identification (MIDI) system, version 6.0 and the ACTINO6 database (Landwehr et al., 2018).

## RESULTS

### *Analysis of 123 German soil samples and identification of a roseoflavin producing* Streptomyces *strain*

**FIGURE 2:**
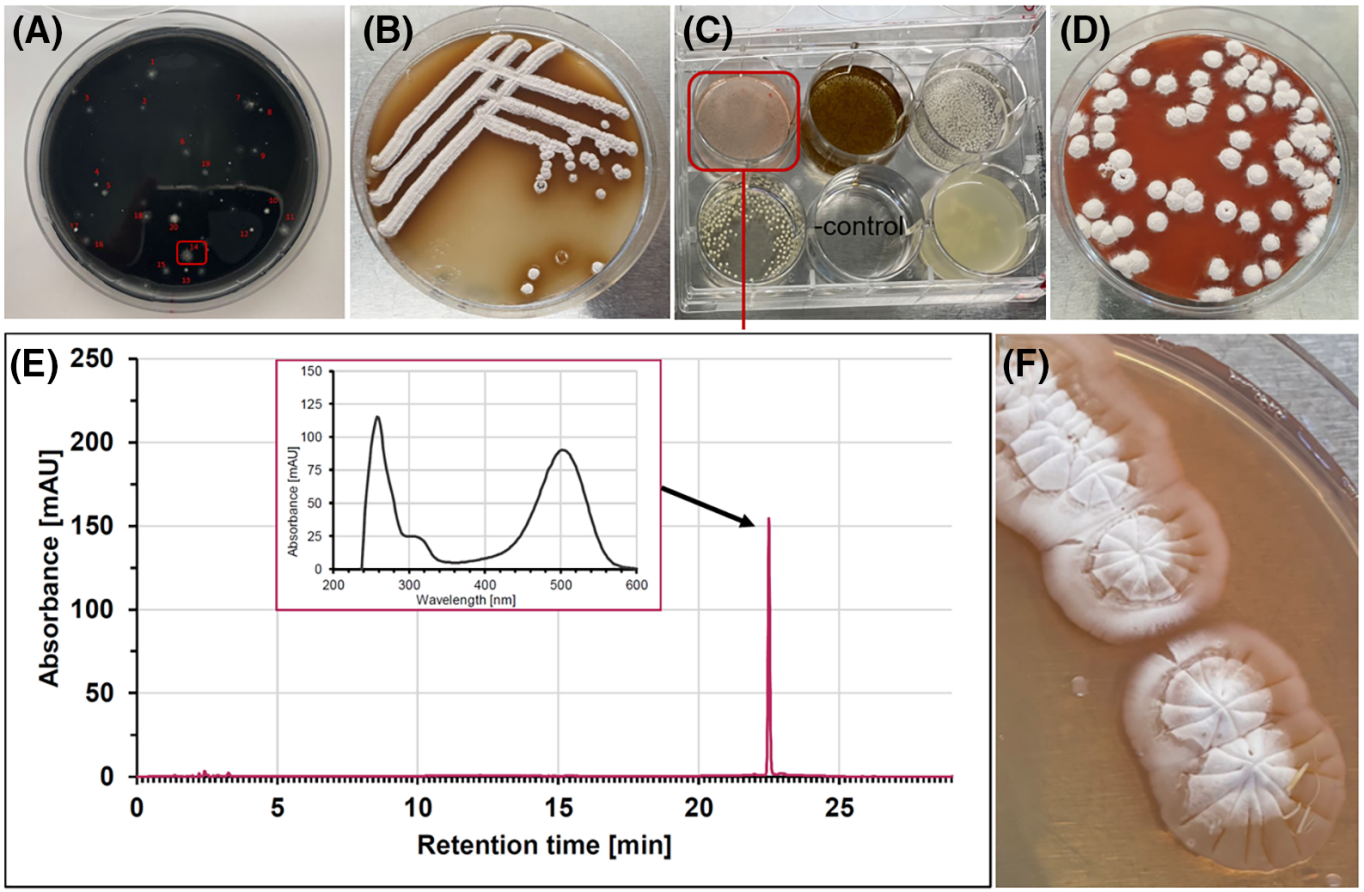
Isolation of a novel roseoflavin producer. Soil samples were suspended, diluted and plated on activated charcoal‐vitamin agar. Those colonies which showed the typical mycelial growth of *Streptomyces* species (A) were transferred to mannitol‐soy‐(MS)‐agar (B) which supports sporulation of *Streptomyces* species. The white, flat colony with jagged edges which in (A) is boxed in red was streaked on MS‐agar to generate isolated colonies and spores (B). Spores from isolated colonies on MS‐agar (such as the one shown in B) were transferred to a liquid growth medium (6‐well plate) containing yeast extract and soluble starch (YS broth) a medium known to support roseoflavin production (C). One of the cultures turned reddish (red box in C) and this isolate tentatively was named ‘*Streptomyces* 14.2’. The colony boxed in red in (A) was also streaked on a YS‐plate (D) and roseoflavin release (red colour) was also observed on this solid growth medium (uninoculated YS is slightly yellowish). The supernatant of the culture in the left upper row (C, see red box) was analysed by HPLC coupled to a photometer and compared to a roseoflavin standard (E). The retention time and the spectrum of the red compound perfectly matched the roseoflavin standard. A fresh culture of isolate *Streptomyces* 14.2 growing on YS agar is about to release roseoflavin (faint reddish colour) (F).

**FIGURE 3:**
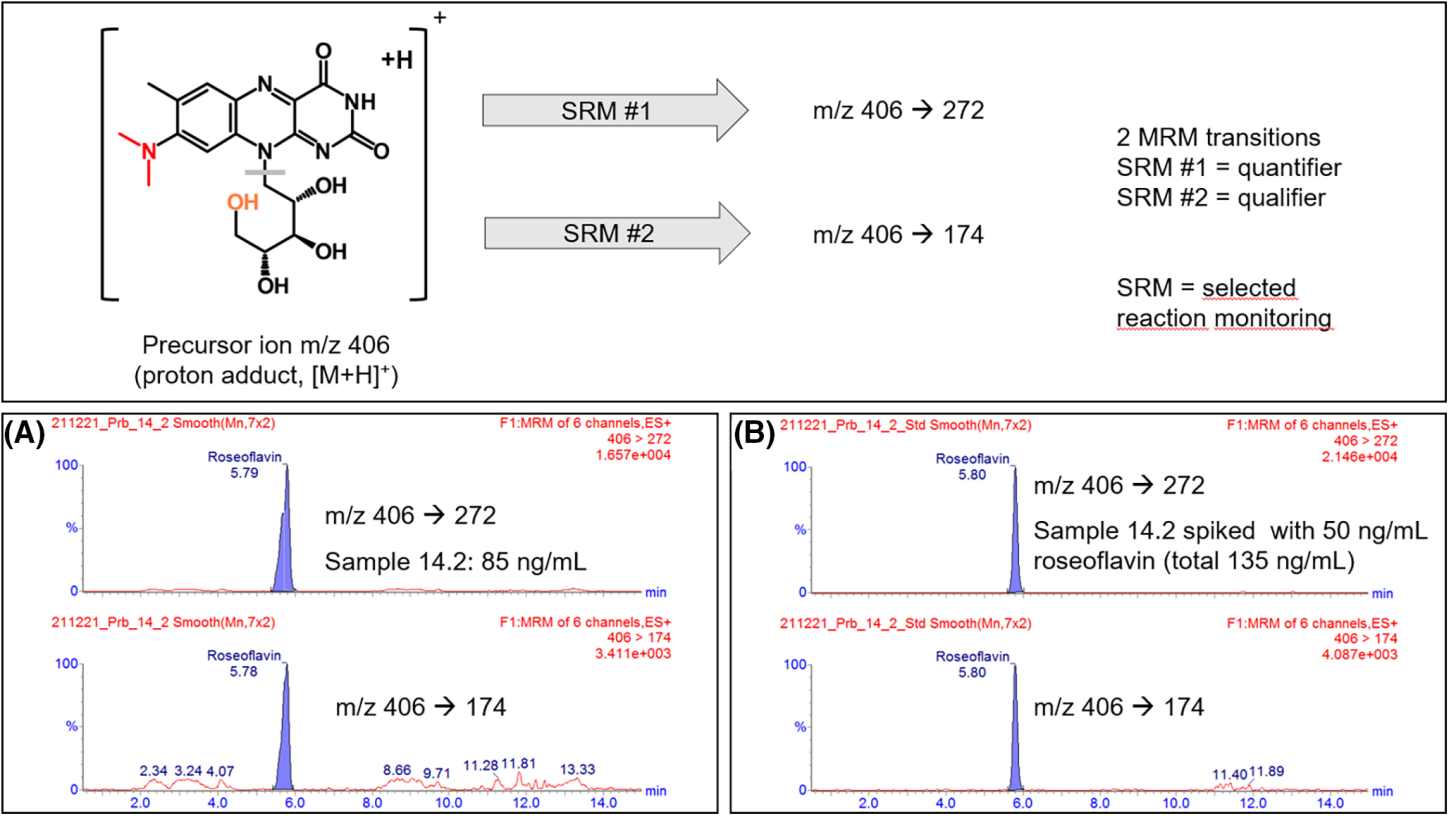
Mass spectrometry confirmed that the red compound (see Figure 2E) was roseoflavin. Electrospray ionisation liquid chromatography tandem mass spectrometric (ESI LC–MS/MS) analysis of the supernatant of a *Streptomyces* 14.2 culture growing on YS (‘Sample 14.2’; A) and a roseoflavin‐fortified ‘Sample 14.2’ (B) confirms that *Streptomyces* 14.2 synthesises roseoflavin. The ESI source was operated in positive mode. Two multiple reaction monitoring (MRM) transitions (m/z 406‐>272 as quantifier and 406‐>174 as qualifier) were used for additional confirmation. They grey bar in the roseoflavin structure (top panel) indicates the site of fragmentation.

We collected 123 soil samples from different locations in Germany (Mannheim, Karlsruhe, Berlin, Koblenz, Aachen, Landshut and Bietigheim‐Bissingen) and generated suspensions. Dilutions of these suspensions were plated on charcoal‐vitamin agar (CV‐agar) and the plates were incubated for 1–2 weeks at 30°C under oxic conditions. CV is selective for bacteria of the genus *Streptomyces*. Colonies showing the typical mycelial growth of *Streptomyces* species (Figure 2A) were transferred to the richer mannitol‐soy‐agar (MS‐agar), which supports sporulation of Streptomycetes. Using this growth medium, colonies appeared following incubation for >3 days. Spores were visible after incubation of the plates for another >10 days at 30°C (Figure 2B). Spores from isolated colonies were transferred to a growth medium containing yeast extract and soluble starch (YS broth) a medium known to support roseoflavin production in the producers *S. davaonensis* and *S. cinnabarinus*. Incubation was carried out in 6‐well cell culture plates on a conventional laboratory shaker for 1 week at 30°C (Figure 2C). One out of 123 cultures (the one which was obtained from ‘Fritz Schloss Park’ in the German capital Berlin; 52°31′45.4″ N13°21′35.9″ E) turned reddish (‘isolate 14.2’) (Figure 2C). We suspected that the reddish colour was due to roseoflavin. Isolate 14.2 was grown on YS agar for 2 weeks and the intense red colour indicated roseoflavin formation as well (Figure 2D). HPLC and UV/Vis spectral analysis of the supernatant of a liquid culture of ‘isolate 14.2’ strongly suggested that the red compound was roseoflavin (Figure 2E). Analysis by HPLC/MS and comparison to a roseoflavin standard confirmed this finding (Figure 3A,B). Growth of *Streptomyces* species in liquid media is characterised by complex mycelial particles (pellets) and such cell pellets were observed when growing the isolate 14.2 in YS broth (Figure S1). Sequencing the 16S rRNA gene confirmed that the novel isolate belonged to the genus *Streptomyces* (Figure S2). When comparing the variable regions of the 16S rRNA gene from *S. davaonensis* and *S. cinnabarinus* to the corresponding 16S rRNA nucleotide sequence from the novel isolate (‘*Streptomyces* 14.2’) 22 different nucleotides were found (Figure S2). The experiments described in this section were repeated twice with soil samples taken 6 months later and 1 year later from the same location (‘Fritz Schloss Park’) (Figure S3) and in both cases roseoflavin producers with an identical 16S rRNA gene as present in *Streptomyces* 14.2 could be isolated (Figure S2).

### *Growth and roseoflavin production by* Streptomyces *14.2*

**FIGURE 4:**
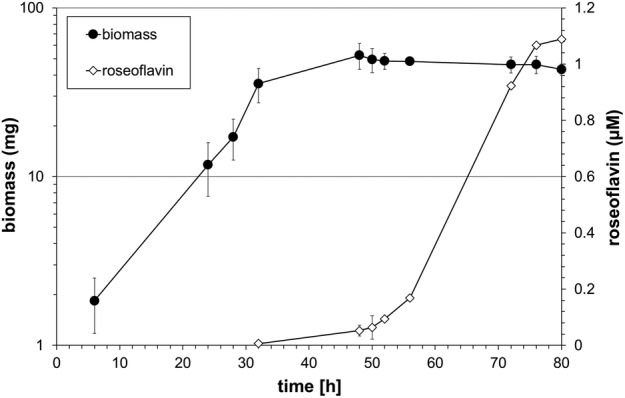
Growth and roseoflavin production of *Streptomyces* 14.2 on YS broth. About 50,000 spores of *Streptomyces* 14.2 were used to inoculate YS. The liquid cultures were aerobically grown at 30°C for 80 h. Dry mass (mg ± SD, *n* = 3) and roseoflavin production (μM ± SD, n = 3) were determined at indicated times. As with *Streptomyces davaonensis* and *Streptomyces cinnabarinus*, roseoflavin synthesis occurs with *Streptomyces* 14.2 in the stationary phase of growth. *S. davaonensis* typically produces less roseoflavin (about 70%) when compared to *Streptomyces* 14.2 whereby *S. cinnabarinus* under laboratory conditions produces about twice as much.

*Streptomyces* 14.2 was grown in YS broth and roseoflavin production was monitored by HPLC (Figure 4). Following 80 h of incubation at 30°C about 1.1 μM roseoflavin was detected in the culture supernatant. The supernatant of a similar *Streptomyces* 14.2 culture was chromatographed on a preparative C18 reversed phase HPLC column to separate roseoflavin from other antibiotics which may be produced by *Streptomyces* 14.2 when grown on YS. The roseoflavin containing fractions (showing a typical absorption maximum at 509 nm) were concentrated and tested in an agar diffusion assay using *Bacillus subtilis* 168 (DSM23778) as an indicator bacterium. A clear zone of inhibition confirmed the antibiotic activity of roseoflavin isolated from *Streptomyces* 14.2 (Figure S4) (Mora‐Lugo et al., 2019).

### *Whole genome sequencing of* Streptomyces *14.2 reveals an unusual chromosomal arrangement and the presence of the roseoflavin biosynthetic genes rosA, rosB and rosC*

A spore suspension was generated from *Streptomyces* 14.2, diluted and spread on MS agar with the goal to not have more than approximately 10 colonies per plate. DNA was extracted from a liquid culture derived from a single, well isolated colony and sequenced. Analysis of the assembly of the first sequencing run suggested that the core chromosome was about 5.675 Mbp in size and that two unusually large (identical) inverted terminal repeats (ITRs, 2476 kbp) were present left and right with about 1.6× the coverage of the core chromosome (Figure S5A,B). In addition, a third small contig of 104 kbp was found that connected to the core chromosome only on one side and had a significantly lower read coverage (0.3×) than the core chromosome. To confirm this unusual genomic structure the whole genome sequencing experiment was repeated with gDNA isolated from a single colony, derived from two passages of spore suspensions. In this case, the assembly resulted in a similar chromosomal arrangement with a larger core (7130 kbp) while the ITRs were shorter 1024 kbp (Figure S5). Again, a small, underrepresented contig was found, albeit missing about 2 kbp at its terminus (102 kbp, 0.3× relative coverage). The coverage distribution indicated that mycelial cells of *Streptomyces* 14.2 carry 2–3 copies of the chromosome, one of which consists of one copy of the ITR, the core and the small contig (henceforth designated as illegitimate terminal end (ITE)), while the other 1–2 are built as found in other *Streptomyces* genomes, that is, a core with ITRs on both ends. This arrangement seems to be unstable, resulting in different ITR lengths in the two cultivations as well as 2 kbp missing at the end of the ITE. Analysis of the putative chromosomal breakpoints or junctions revealed the presence of a 1.4 kbp repetitive element encoding an IS110 family transposase, which may be the cause of this chromosomal rearrangement. To the best of our knowledge, this is the first description of such a heterogenic genomic arrangement in Streptomycetes. Due to the genomic instability, the number of genes present in the *Streptomyces* 14.2 genome is variable. The submitted version derived from a single colony contains 897 genes per ITR, 6190 genes in the core and 107 genes located on the ITE (GenBank accession No. CP115392– CP115394.1; BioProject PRJNA868580). The genome of the deposited sequence of *Streptomyces* 14.2 has a G + C content of 71.47%. The automatic annotation of the genome of *Streptomyces* 14.2 revealed that the roseoflavin biosynthetic genes *rosA*, *rosB* and *rosC* in addition to a high number of riboflavin biosynthetic genes were present.

### *Heterologous expression of the genes rosA, rosB and rosC from* Streptomyces *14.2 in* Escherichia coli *and enzymatic analysis of the overproduced enzymes*

To confirm that the genes *rosA*, *rosB* and *rosC* from *Streptomyces* 14.2 indeed are responsible for roseoflavin synthesis (Figure 1) they were overexpressed separately in three different *E. coli* strains using a T7 RNA polymerase‐based system. Since the enzymes RosA, RosB and RosC catalyse unique biochemical reactions and are not present in *E. coli* the overproduced *Streptomyces* 14.2 proteins RosA, RosB and RosC could be tested in cell‐free extracts of overproducing *E. coli* strains without purification. All enzymes carried out the expected function i.e. the conversion of FMN to 8‐demethyl‐8‐amino‐riboflavin‐5′‐phosphate (AFP) in the presence of glutamate (RosB; Figure S6A,B), the dephosphorylation of AFP to 8‐demethyl‐8‐amino‐riboflavin (AF) (RosC; Figure S7A,C) and the two methylation reactions, which convert AF to roseoflavin (RosA; Figure S8A,B).

### *The roseoflavin biosynthetic genes in* Streptomyces *14.2 are organised in a similar way as in* S. davaonensis *and* S. cinnabarinus

A 120 kbp subgenomic *Streptomyces* 14.2 fragment harbouring the *ros*‐genes was compared to the corresponding genes in *S. davaonensis* and *S. cinnabarinus* (Figure S9). In the three different roseoflavin‐producing species, the *ros*‐genes are distributed in two gene clusters separated by about 50–65 kbp. The *rosB* cluster is highly conserved and comprises the genes *rosG*, *ribE2*, *ribX*, *ribY* and *rosB*. The *rosA* cluster is not as strongly conserved and comprises 10 genes in *S. davaonensis* and *S. cinnabarinus* and 11 genes in *Streptomyces* 14.2. The function of this additional gene in *Streptomyces* 14.2 is unknown. The genes indicative for roseoflavin biosynthesis, *rosA* and *rosC*, are present in all three *rosA* clusters. Many of the gene products of the two *ros*‐clusters are highly similar and sequence comparisons are shown in Table 1. BLASTp analyses identified roseoflavin biosynthetic enzymes in two additional isolates, *Streptomyces* sp. HUAS 2–6 and *Streptomyces* sp. NBRC 14336 and comparisons to these sequences are shown as well (Table 1). The latter two isolates have not been deposited in public strain collections and thus flavin production by these bacteria could not be examined.

**TABLE 1:** The listed *Streptomyces* species are the only species which contain gene products (in bold) involved in roseoflavin metabolism (see Figure 1). Sequence identities (in %) above 90% are shown in red (*Streptomyces davaonensis* was set to 100%). Genes/enzymes in bold are either missing or represent additional functions not found in the other *Streptomyces* species analysed in this table.

*S. davao‐nensis*		*S. cinnabarinus*	*Streptomyces* 14.2	*Streptomyces* NBRC	*Streptomyces* HUAS
***rosA* cluster**					
RS39765 (WP_015662730.1)	100	98.39 (WP_269657273.1)	94.02 (WBO76198.1)	93.79 (WP_285512165.1)	89.89 (WP_270086817.1)
RS39770 (WP_015662731.1)	100	99.02 (WP_269657272.1)	94.29 (WBO76197.1)	94.09 (WP_285512164.1)	87.99% (WP_270086818.1)
RS39775 (WP_015662732.1)	100	91.68 (WP_269657271.1)	83.73 (WBO76196.1)	84.09 (WP_285512163.1)	73.14 (WP_270086819.1)
RS39780 (WP_015662733.1)	100	96.70 (WP_269657270.1)	89.01 (WBO76195.1)	89.09 (WP_285512162.1)	82.42 (WP_270086820.1)
RS39785 (WP_015662734.1)	100	92.86 (WP_269657269.1)	87.19 (WBO76194.1)	87.68 (WP_285512161.1)	71.80 (WP_270086821.1)
RS39790 (WP_015662735.1)	100	99.32 (WP_269657268.1)	93.01 (WBO76193.1)	93.01 (WP_285512160.1)	90.91 (WP_270086822.1)
**RosA** (WP_015662736.1)	100	95.97 (WP_269657267.1)	88.15 (WBO76192.1)	88.15 (WP_285512159.1)	**No similar gene**
**RosC** (WP_015662737.1)	100	97.75 (WP_269657266.1)	94.59 (WBO76191.1)	94.59 (WP_285512158.1)	85.20 (WP_270086823.1)
RS39805 (WP_015662738.1)	100	93.46 (WP_269657265.1)	84.29 (WBO76190.1)	84.29 (WP_285512157.1)	**No similar gene**
	**No similar gene**	**No similar gene**	(WBO76189.1)	**No similar gene**	**No similar gene**
RS39810 (WP_015662739.1)	100	94.90 (WP_269657264.1)	84.62 (WBO76188.1)	85.90 (WP_285512156.1)	**No similar gene**
***rosB* cluster**					
**RosG** (WP_015662690.1)	100	95.94 (WP_269657320.1)	85.85 (WBO76237.1)	85.66 (WP_285512205.1)	77.71 (WP_270086806.1)
**RibE2** (WP_015662691.1)	100	98.73 (WP_269657319.1)	89.24 (WBO76236.1)	89.24 (WP_285512204.1)	85.26 (WP_270086807.1)
**RibX** (WP_015662692.1)	100	98.39 (WP_269657318.1)	94.42 (WBO76235.1)	94.40 (WP_285512203.1)	**No similar gene**
**RibY** (WP_015662693.1)	100	94.60 (WP_269657317.1)	85.51 (WBO76234.1)	85.51 (WP_285512202.1)	70.87 (WP_270086808.1)
**RosB** (WP_015662694.1)	100	99.22 (WP_269657316.1)	96.50 (WBO76233.1)	96.50 (WP_285512201.1)	89.49 (WP_270086809.1)

*Note*: Identities at the amino acid level in %. Genbank IDs are given in brackets.

### Whole genome analysis of the novel isolate reveals that an unusually high number of riboflavin biosynthetic genes is present

In a previous work, genome annotations of 256 different *Streptomyces* species were analysed with regard to the number of genes predicted to be involved in riboflavin biosynthesis (Kißling et al., 2020). Seven genes, that is, their corresponding gene products are necessary to synthesise riboflavin from GTP and ribulose 5′‐phosphate (Fischer et al., 2004), however, in *S. davaonensis* and *S. cinnabarinus* > 21 riboflavin biosynthetic genes were found (Kißling et al., 2020). These roseoflavin producers apparently have a high catalytic capacity to synthesise riboflavin, the immediate precursor of FMN, which in turn is the starting point for roseoflavin biosynthesis (Figure 1) (Kißling et al., 2020). The finding that the novel roseoflavin producer *Streptomyces* 14.2 contains 18 riboflavin biosynthetic genes is in line with this idea that roseoflavin releasing organisms depend on increased cellular riboflavin levels (Mora‐Lugo et al., 2019). Expression of two of these riboflavin biosynthetic genes in strain 14.2 are controlled by FMN riboswitches. One FMN riboswitch is located immediately upstream of the *Streptomyces* 14.2 operon *ribE1MAB5H*. Surprisingly, the aptamer portion of this FMN riboswitch (134 nucleotides) is identical to the aptamer portion of the corresponding *ribE1MAB5H* FMN riboswitch of *S. davaonensis* (Figure S10) (Pedrolli et al., 2012). In the *S. davaonensis ribE1* FMN riboswitch a specific nucleotide is present, which confers roseoflavin resistance. The same nucleotide is present in *Streptomyces* 14.2 (the aptamer is identical) and we conclude that this *ribE1* FMN riboswitch also confers roseoflavin resistance to this roseoflavin producer (Figure S10) (Pedrolli et al., 2012). The second FMN riboswitch (136 nucleotides) is present upstream of the riboflavin biosynthetic gene *ribB9* of *Streptomyces* 14.2. The corresponding aptamer portion is again identical to the one present in the *S. davaonensis ribB9* FMN riboswitch (Pedrolli et al., 2012). This riboswitch was found to be roseoflavin (RoFMN)‐sensitive and we conclude that the *Streptomyces* 14.2 *ribB9* FMN riboswitch is RoFMN‐sensitive as well. As in *S. davaonensis* (Jankowitsch et al., 2012), a gene encoding an additional riboflavin synthase (*ribE2*) is part of the *Streptomyces* 14.2 roseoflavin biosynthetic *rosB* gene cluster (Figure S9). The gene product RibE2 is very similar to archaeal riboflavin synthases, which do not show similarity to the corresponding eubacterial enzymes (Fischer & Bacher, 2005) and, as for *S. davaonensis*, we suggest that *ribE2* in *Streptomyces* 14.2 is a result of horizontal gene transfer (Jankowitsch et al., 2012).

### Streptomyces *14.2 represents a novel species*

Only one 16S rRNA gene sequence (present in six copies scattered over the chromosome) was detected in *Streptomyces* 14.2 using the ContEst16S tool (www.ezbiocloud.net; accessed on 8 April 2023), indicating that the isolate was pure. A phenetic analysis (EzBioCloud database) employing the 16S rRNA of *Streptomyces* 14.2 revealed that strain 14.2 was most closely related to *Streptomyces nigra* 452^T^ (99.38%), *Streptomyces albogriseolus* NRRL B‐1305^T^ (99.17%), and *Streptomyces iakyrus* NRRL ISP‐5482^T^ (99.10%). The phylogenetic relationships of strain 14.2 to other *Streptomyces* species based on analysis of a sequence alignment resulting from concatenation of five house‐keeping genes (*atpD*, *gyrB*, *recA*, *rpoB* and *trpB*; see Table S1) is shown in Figure S11. Strain 14.2 was located in the same branch as *S. cupreus* PSKA01^T^ which is not a roseoflavin producer. This relationship was supported by a high bootstrap value and also by maximum‐likelihood and maximum‐parsimony analyses. MLSA evolutionary distances are shown in Table S2. Strain 14.2 exhibited an MLSA distance greater than 0.007 (corresponding to 70% DNA–DNA similarity) indicating that this strain can be distinguished from other *Streptomyces*.

The phylogenomic tree (Figure S12) suggests with a very high support value that strain *Streptomyces* sp. 14.2 is located in one clade with *S. cinnabarinus* NRRL B‐12382^T^, *S. davaonensis* JCM 4913^T^ and *Streptomyces cupreus* PSKA01^T^, indicating that strain 14.2 was most closely related to these *Streptomyces* type strains. The DNA–DNA hybridisation (dDDH) similarities between the genome of *Streptomyces* sp. 14.2 and *S. cinnabarinus* NRRL B‐12382^T^, *S. davaonensis* JCM 4913^T^ and *Streptomyces cupreus* PSKA01^T^ were 36.1%, 35.6% and 34.2%, respectively (Table S3). These values are below the 70% threshold indicating that these bacteria represent different species (Moore et al., 1987). The pairwise orthologous average nucleotide identity (orthoANI) values of the genomes of strain 14.2^T^ and its most closely related type strains were 88.22% for *S. cinnabarinus* NRRL B‐12382^T^, 88.45% for *S. davaonensis* JCM 4913^T^, and 87.78% for *S. cupreus* PSKA01^T^ (Table S3). These values were below the threshold of 95% as well indicating that these bacteria represent different species (Chun et al., 2018).

To generate further evidence that strain 14.2 represents an own species, a series of taxonomic analyses were carried out. In addition to genotypic studies (see above) strain 14.2 could also be discriminated from its closely related *Streptomyces* type strains by a series of phenotypic properties, which are described in the supplement (Tables S4‐S8 and Figures S13–S16). In summary, strain 14.2 represents a novel species in the genus *Streptomyces*, for which the name *Streptomyces berlinensis* sp. nov. is proposed. The name refers to the sampling site, the German capital Berlin.

## DISCUSSION

Not all microbial species are found everywhere. Some species can thrive, or at least tolerate, a broad range of environmental conditions and are more likely to be ubiquitous. Other species can only persist under a very specific set of environmental conditions and subsequently have far more restricted ranges. The factors that explain these differences in environmental and/or geographical distributions across microbial species remain unresolved (Barberán et al., 2014). The red antibiotic roseoflavin can easily be detected in liquid cultures and the roseoflavin biosynthetic enzymes RosA, RosB and RosC have been found in three valid species only (including this work). Thus, roseoflavin synthesis could serve as a model to study the distribution of specific biosynthetic capacities in soil populations. In this context, it is important to mention that a recent work described the isolation of *S. davaonensis* (but not of a novel roseoflavin‐producing species) from a termite queen captured in the suburb of Lanxi City, Zhejiang Province, China (Zhou et al., 2021).

When we use the primary structure of the key enzyme of roseoflavin biosynthesis RosB from *S. davaonensis* and carry out a BLASTp search we find highly similar proteins (>89% identities at the amino acid level over the complete sequence) only in *S. davaonensis* (100%), *S. cinnabarinus* (99.22%), *S. berlinensis* (96.50%), *Streptomyces* sp. HUAS 2–6 (89.49%) and *Streptomyces* sp. NBRC 14336 (96.50%) (Table 1). As an FMN binding enzyme RosB has a weak correlation to members of the flavodoxin superfamily of NAD(P)H:FMN‐dependent reductases (Schwarz et al., 2016) and we thus find other hits as well (<40% identities at the amino acid level), however, these gene products are not involved in roseoflavin biosynthesis since they lack the highly specialised C‐terminus (Schwarz et al., 2016). When the unique C‐terminal part of RosB (52 amino acids) is used for a BLASTp search hits are only found in *S. davaonensis*, *S. cinnabarinus*, *S. berlinensis*, *Streptomyces* sp. HUAS 2–6 and *Streptomyces* sp. NBRC 14336. Notably, the latter two isolates have not been analysed with regard to roseoflavin synthesis. *Streptomyces* sp. HUAS 2–6 contains proteins, which are highly similar to RosC and RosB (identities at the amino acid level > 85%) but not to RosA (Table 1). We thus predict that *Streptomyces* sp. HUAS 2–6 is a producer of 8‐demethyl‐8‐amino‐riboflavin (AF) (but not of roseoflavin) since the final dimethyltransferase RosA, which converts AF to roseoflavin (Figure 1), apparently is not present in this organism. Provided the deposited genomic sequence of *Streptomyces* sp. HUAS 2–6 is complete this organism would be the first isolate to produce AF which was shown to have antibiotic activity as well (Matern et al., 2016; Pedrolli et al., 2011). *Streptomyces* sp. NBRC 14336 contains proteins, which are highly similar to RosC, RosB and RosA (identities at the amino acid level > 88%) and we suggest that this isolate is a roseoflavin producer (Table 1). We were not able to test this as the strain is not available.

*S. davaonensis* and *S. cinnabarinus* were both isolated decades ago from Asian soil samples whereby the production of roseoflavin by *S. cinnabarinus* was not recognised at the time of isolation (Jankowitsch et al., 2012). With the objective to isolate unknown roseoflavin‐producing Streptomycetes, we collected soil samples from different places in Germany. The analysis of 123 soil samples was sufficient to discover *Streptomyces berlinensis*, a novel roseoflavin‐producing species. The three species are closely related, although they were isolated from very distant geographical locations.

## AUTHOR CONTRIBUTIONS

**Jimmy Jonathan Liunardo:** Investigation (equal). **Sebastien Messerli:** Investigation (equal). **Ann‐Kathrin Gregotsch:** Investigation (equal). **Sonja Lang:** Investigation (equal). **Kerstin Schlosser:** Investigation (equal). **Christian Rückert‐Reed:** Investigation (equal). **Tobias Busche:** Investigation (equal). **Jörn Kalinowski:** Investigation (equal). **Martin Zischka:** Investigation (supporting). **Philipp Weller:** Investigation (supporting). **Imen Nouioui:** Investigation (supporting). **Meina Neumann‐Schaal:** Investigation (equal). **Chandra Risdian:** Investigation (equal); writing – review and editing (supporting). **Joachim Wink:** Investigation (equal); writing – review and editing (supporting). **Matthias Mack:** Conceptualization (lead); formal analysis (lead); funding acquisition (lead); project administration (lead); supervision (lead); writing – original draft (lead); writing – review and editing (lead).

## CONFLICT OF INTEREST STATEMENT

The authors declare no conflict of interest.

## Supporting information


**DATA S1:** Supporting Information.

## Data Availability

The data that supports the findings of this study are available in the supplementary material of this article.
