# Supplementary material for: Isolation, characterisation and description of the roseoflavin producer Streptomyces berlinensis sp. nov
Source: Environ Microbiol Rep. 2024 Apr 23;16(2):e13266. doi: 10.1111/1758-2229.13266 (PMC11039241; doi:10.1111/1758-2229.13266)
Supplement: Supplementary file 1 — DATA S1: Supporting Information. [file EMI4-16-e13266-s001.docx]

**Isolation, characterization and description of the roseoflavin producer *Streptomyces berlinensis* sp. nov.**

Jimmy Jonathan Liunardo^a^, Sebastien Messerli^a^, Ann-Kathrin Gregotsch^a^, Sonja Lang^a^, Kerstin Schlosser^a^, Christian Rückert-Reed^b,c^, Tobias Busche^b^, Jörn Kalinowski^c^, Martin Zischka^d^, Philipp Weller^d^, Imen Nouioui^e^, Meina Neumann-Schaal^e^, Chandra Risdian^f,g^, Joachim Wink^f,h^ and Matthias Mack^a^#

^a^Institute for Technical Microbiology, Department of Biotechnology, Mannheim University of Applied Sciences, 68163 Mannheim, Germany

^b^Medical School East Westphalia-Lippe, Bielefeld University, 33615 Bielefeld, Germany

^c^Technology Platform Genomics, Center for Biotechnology, Bielefeld University, 33615 Bielefeld, Germany

^d^Institute for Instrumental Analytics and Bioanalytics, Department of Biotechnology, Mannheim University of Applied Sciences, 68163 Mannheim, Germany

^e^Leibniz-Institute DSMZ-German Collection of Microorganisms and Cell Cultures, 38124 Braunschweig, Germany

^f^Department of Microbial Strain Collection, Helmholtz Centre for Infection Research, 38124 Braunschweig, Germany

^g^Research Center for Applied Microbiology, National Research and Innovation Agency (BRIN), 40135 Bandung, Indonesia

^h^German Centre for Infection Research (DZIF), Partner Site Hannover-Braunschweig, 38124 Braunschweig, Germany

Running Head: A novel roseoflavin producer

#Address correspondence to Matthias Mack, m.mack@hs-mannheim.de, ORCID ID: 0000-0002-7753-2422, Scopus Author ID: 57210454909

**Supporting tables S1-S9, figures S1-S17 and references of the supplement**

**Table S1** House-keeping gene sequences of *Streptomyces* strains employed in the present study to analyze phylogenetic relationships.

| **Strain** | ***atpD*** | ***gyrB*** | ***recA*** | ***rpoB*** | ***trpB*** |
| --- | --- | --- | --- | --- | --- |
| Strain 14.2 | CP115393: region 4493588-4495024 | CP115393: region 3052663-3054678 | CP115393: region 4909218-4910342 | CP115393: region 3796403-3799888 | CP115393: region 995902-997191 |
| *S. iakyrus* NRRL B-3317^T^ | KT384600 | KT384949 | KT385298 | KT388920 | KT389269 |
| *S. albogriseolus* NRRL B-1305^T^ | KT384453 | KT384802 | KT385150 | KT388772 | KT389122 |
| *S. nigra* MG572975^T^ | CP029043 : region 5139758-5141194 | CP029043 : region 3805356-3807440 | CP029043 : region 5614335-5615459 | CP029043 : region 4445687-4449172 | CP029043 : region 1844966-1846249 |
| *S. flaveolus* JCM 4032^T^ | BMRV01000002: region 70323-71771 | BMRV01000004: region 379584-381668 | BMRV01000009: region 31914-33050 | BMRV01000002: region 751794-755279 | BMRV01000001: region 566656- 567942 |
| *S. cupreus* PSKA01^T^ | NZ_JACMSF010000032: region 74643-76079 | NZ_JACMSF010000038: region 85149-87275 | NZ_JACMSF010000044: region  18026-19150 | NZ_JACMSF010000005: region 53292-56777 | NZ_JACMSF010000067: region 21019-22302 |
| *S. massasporeus* NRRL B-3300^T^ | KT384636 | KT384985 | KT385336 | KT388956 | KT389305 |
| *S. ferrugineus* CCTCC AA2014009^T^ | CP063373: region 8201512- 8202948 | CP063373: region 236202- 238262 | CP063373: region 7686387-7687514 | CP063373: region 9207294- 9210779 | CP063373: region 2570442- 2571728 |
| *S. spinoverrucosus* NBRC 14228^T^ | NZ_BJND01000053: region 34282-35718 | NZ_BJND01000047: region 76927-78987 | NZ_BJND01000069: region 5740-6864 | NZ_BJND01000017: region 177067-180552 | NZ_BJND01000012: region 75197-76483 |
| *S. griseosporeus* NRRL B-12498^T^ | KT384584 | KT384933 | KT385282 | KT388904 | KT389253 |
| *S. davaonensis* JCM 4913^T^ | HE971709: region 3455438- 3456874 | HE971709: region 5040088-5042157 | HE971709: region 3035230-3036348 | HE971709: region 4206204-4209689 | HE971709: region 7189492-7190781 |
| *S. cinnabarinus* DSM 40467^T^ | CP114413: region 6071917-6073353 | CP114413: region 4603638-4605683 | CP114413: region 6548465-6549589 | CP114413: region 5311139-5314624 | CP114413: region 2485814-2487097 |
| *S. cyaneochromogenes* MK-45^T^ | CP034539: region 6900220-6901656 | CP034539: region 5136701-5138785 | CP034539: region 7440462-7441586 | CP034539: region 6030712-6034197 | CP034539: region 2700920-2702206 |
| *S. chartreusis* NRRL ISP-5085^T^ | KT384507 | KT384856 | KT385204 | KT388826 | KT389176 |
| *S. caeruleatus* NRRL B-24802^T^ | KT384496 | KT384845 | KT385193 | KT388815 | KT389165 |
| *S. violaceochromogenes* NRRL B-5427^T^ | KT384748 | KT385096 | KT385450 | KT389068 | KT389417 |
| *S. coerulescens* AS 4.1597^T^ | FJ406145 | FJ406201 | FJ406257 | FJ406313 | FJ406368 |
| *S. albus* subsp. *albus* NRRL B-2208^T^ | KF528055 | KF528056 | KF528057 | KJ996610 | KF528059 |

**Table S2** Multilocus sequence analysis (MLSA) of streptomycetes closely related to strain 14.2.

| Strain | MLSA (Kimura 2-parameter) distance |
| --- | --- |
| *Streptomyces iakyrus* NRRL B-3317^T^ | 0,0710 |
| *Streptomyces albogriseolus* NRRL B-1305^T^ | 0,0626 |
| *Streptomyces nigra* 452^T^ | 0,0587 |
| *Streptomyces flaveolus* JCM 4032^T^ | 0,0639 |
| *Streptomyces cupreus* PSKA01^T^ | 0,0380 |
| *Streptomyces massasporeus* NRRL B-3300^T^ | 0,0710 |
| *Streptomyces ferrugineus* CCTCC AA2014009^T^ | 0,0608 |
| *Streptomyces spinoverrucosus* NBRC 14228^T^ | 0,0504 |
| *Streptomyces griseosporeus* NRRL B-12498^T^ | 0,0600 |
| *Streptomyces davaonensis* JCM 4913^T^ | 0,0457 |
| *Streptomyces cinnabarinus* DSM 40467^T^ | 0,0427 |
| *Streptomyces cyaneochromogenes* MK-45^T^ | 0,0591 |
| *Streptomyces chartreusis* NRRL ISP-5085^T^ | 0,0547 |
| *Streptomyces caeruleatus* NRRL B-24802^T^ | 0,0626 |
| *Streptomyces violaceochromogenes* NRRL B-5427^T^ | 0,0754 |
| *Streptomyces coerulescens* AS 4.1597^T^ | 0,0530 |
| *Streptomyces albus* subsp. *albus* NRRL B-2208^T^ | 0,1304 |

**Table S3** DNA-DNA hybridization (dDDH) and orthologous average nucleotide identity (orthoANI) analysis of strain 14.2 and closely related *Streptomyces* species.

| **Strain** | **dDDH (%)** | **OrthoANI values (%)** |
| --- | --- | --- |
| *Streptomyces iakyrus* NRRL-ISP 5482^T^ | 26.8 | 83.37 |
| *Streptomyces albogriseolus* JCM 4616^T^ | 25.8 | 82.73 |
| *Streptomyces nigra* 452^T^ | 27.0 | 83.47 |
| *Streptomyces flaveolus* JCM 4032^T^ | 26.8 | 83.53 |
| *Streptomyces cupreus* PSKA01^T^ | 34.2 | 87.78 |
| *Streptomyces massasporeus* JCM 4139^T^ | 26.7 | 83.24 |
| *Streptomyces ferrugineus* CCTCC AA2014009^T^ | 27.9 | 83.97 |
| *Streptomyces spinoverrucosus* NBRC 14228^T^ | 27.1 | 83.48 |
| *Streptomyces griseosporeus* JCM 4766^T^ | 27.0 | 83.66 |
| *Streptomyces davaonensis* JCM 4913^T^ | 35.6 | 88.45 |
| *Streptomyces cinnabarinus* NRRL B-12382^T^ | 36.1 | 88.22 |
| *Streptomyces cyaneochromogenes* MK-45^T^ | 27.8 | 83.83 |
| *Streptomyces chartreusis* ATCC 14922^T^ | 27.2 | 83.58 |
| *Streptomyces caeruleatus* NRRL B-24802^T^ | 27.4 | 83.65 |
| *Streptomyces pilosus* JCM 4403^T^ | 26.1 | 82.85 |
| *Streptomyces matensis* JCM 4277^T^ | 25.9 | 82.69 |
| *Streptomyces thermoviolaceus* subsp. *apingens* JCM 4312^T^ | 25.8 | 83.65 |
| *Streptomyces thermoviolaceus* JCM 4843^T^ | 25.8 | 82.07 |
| *Streptomyces spectabilis* JCM 4308^T^ | 23.0 | 79.67 |

**Table S4** Menaquinone profiles of *Streptomyces* 14.2 and its close phylogenetic neighbors are significantly different.

|  | *Streptomyces* 14.2^T^ | *S. cinnabarinus* DSM 40467^T^ | *S. davaonensis* DSM 101723^T^ |
| --- | --- | --- | --- |
| MK8 H4 | 2.1 | 0.7 | 1.3 |
| MK8 H6 | 1.2 | 0.7 | 0.7 |
| MK8 H8 | Nd | 1.2 | 1.0 |
| MK9 | 0.6 | 1.5 | 1.6 |
| MK9 H2 | 2.8 | 3.9 | 6.2 |
| MK9 H4 | 7.0 | 14.4 | 15.4 |
| MK9 H6 | 45.0 | 26.1 | 35.2 |
| MK9 H8 | 39.5 | 46.2 | 36.1 |
| MK9 H10 | 0.4 | 2.0 | 0.6 |
| MK10 H4 | 0.6 | 1.4 | 1.5 |
| MK10 H6 | 0.8 | 2.0 | 0.4 |
| MK8 H4 | 2.1 | 0.7 | 1.3 |
| MK8 H6 | 1.2 | 0.7 | 0.7 |
| MK8 H8 | Nd | 1.2 | 1.0 |
| MK9 | 0.6 | 1.5 | 1.6 |

**Table S5** Fatty acid profiles of *Streptomyces* 14.2 and its close phylogenetic neighbors *S. cinnabarinus* and *S. davaonensis* are significantly different.

|  | *Streptomyces* 14.2^T^ | *S. cinnabarinus* DSM 40467^T^ | *S. davaonensis* DSM 101723^T^ |
| --- | --- | --- | --- |
| C_15:0_ *iso* | 13.4 | 7.6 | 8.0 |
| C_15:0_ *anteiso* | 17.6 | 17.1 | 15.1 |
| C_15:0_ | - | 7.3 | - |
| C_16:0_ *iso* | 12.7 | 9.8 | 8.6 |
| C_16:1_ cis 9 | 4.9 | 7.3 | 4.9 |
| C_16:0_ | 12.3 | 14.0 | 9.6 |
| C_16:0_ 9? methyl | 4.5 | - | 6.3 |
| C_17:0_ *anteiso* | 13.9 | 12.3 | 23.5 |
| C_17:0_ *iso* | 8.6 | 4.7 | 12.1 |
| C_17:1_ anteiso C | - | - | 4.7 |

Only fatty acids ≥ 4.5% are shown

**Table** **S6** Morphological (ISP media + carbohydrates) and physiological properties of *Streptomyces cinnabarinus* (DSM 40467^T^*), Streptomyces davaonensis* (JCM 4913^T^ = DSM 101723^T^) and *Streptomyces* 14.2 (JS360 = DSM 101724^T^) are different.

|  | *S. cinnabarinus* | *S. davaonensis* | *S.berlinensis* |
| --- | --- | --- | --- |
| **ISP2** |  |  |  |
| Growth | good | good | good |
| colony color | brown red | sand yellow | pastel yellow |
| aerial mycelium | cream | none | telegrey |
| soluble pigment | salmon pink | none | salmon orange |
| **ISP3** |  |  |  |
| Growth | good | good | good |
| colony color | salmon pink | light pink | salmon orange |
| aerial mycelium | cream | signal white | signal white |
| soluble pigment | salmon pink | beige red | none |
| **ISP4** |  |  |  |
| Growth | good | good | good |
| colony color | salmon pink | red orange | saffron yellow |
| aerial mycelium | cream | beige grey | signal white |
| soluble pigment | salmon pink | none | beige red |
| **ISP5** |  |  |  |
| Growth | good | good | good |
| colony color | salmon pink | black red | pastel yellow |
| aerial mycelium | sparse | beige grey | signal white |
| soluble pigment | none | none | beige red |
| **ISP6** |  |  |  |
| Growth | good | decreased | good |
| colony color | olive drab | green brown | brown grey |
| aerial mycelium | none | none | none |
| soluble pigment | olive drab | olive brown | olive brown |
| **ISP7** |  |  |  |
| Growth | good | good | good |
| colony color | Salmon pink | black red | pastel yellow |
| aerial mycelium | sparse | beige grey | signal white |
| soluble pigment | none | olive brown | pastel yellow |

**Table S7** The physiological properties of 1, *S. davaonensis* JCM 4913 = DSM 101723^T^, 2, *S. cinnabarinus* DSM 40467^T^ and, 3, *Streptomyces* 14.2 are different.

|  | **1** | **2** | **3** |
| --- | --- | --- | --- |
| **APIZYME® enzyme tests** |  |  |  |
| Phosphatase alkaline | + | + | + |
| Esterase (C4) | + | + | + |
| Esterase lipase (C8) | + | + | + |
| Lipase (C14) | + | + | - |
| Leucine arylamidase | + | + | + |
| Valine arylamidase | + | + | + |
| Cystine arylamidase | + | + | + |
| Trypsin | + | + | + |
| Chymotrypsin | - | - | (+) |
| Phosphatase acid | + | + | + |
| Naphtol-AS-BI-phosphohydrolase | + | + | + |
| α galactosidase | - | - | - |
| Β-galactosidase | + | + | + |
| β glucuronidase | - | - | - |
| α glucosidase | + | + | + |
| β glucosidase | + | + | + |
| N-acetyl-β-glucoseamidase | + | - | + |
| α mannosidase | + | + | + |
| α fucosidase | - | + | - |
| **Carbon source utilization** |  |  |  |
| Glucose | + | + | + |
| Arabinose | + | + | + |
| Sucrose | + | + | + |
| Xylose | + | - | + |
| Inositol | + | + | + |
| Mannose | + | + | + |
| Fructose | + | + | + |
| Rhamnose | + | + | + |
| Raffinose | + | + | - |
| Cellulose | - | + | - |

**Table S8** Phenotypic properties that distinguish strain 14.2 from closely related *Streptomyces* species.

| **Characteristics** | **1** | **2*** | **3**** | **4**** | **5^***^** | **6** | **7** |
| --- | --- | --- | --- | --- | --- | --- | --- |
| **Spore chains** | Slightly rough spores; Rectus-Flexibilis | No spore chain | Smooth; Rectus-Flexibilis | ND | Smooth; spiral | Slightly rough spores; Rectus-Flexibilis^#^ | Slightly rough spores; Rectus-Flexibilis^#^ |
| **Growth on ISP2 Medium** |  |  |  |  |  |  |  |
| Substrate mycelium colour | Brown red | White | Yellow/ brown | Black olive | Metallic copper/ tan | Brown red | Sand yellow |
| Aerial mycelium colour | Cream | White | Grey | Olive green | Metallic copper/ tan | Cream | - |
| Soluble pigment(s) | Salmon pink | - | - | - | ND | Salmon pink | - |
| **Growth on sole carbon source** |  |  |  |  |  |  |  |
| Fructose | + | + | ND | + | + | + | + |
| Raffinose | - | ND | ND | + | + | + | + |
| Sucrose | + | + | ND | + | - | + | + |
| **Major fatty acids** | C15:0 *iso*;  C15:0 *anteiso*;  C16:0 *iso*; C16:0; C17:0 *anteiso* | C15:0 *iso*; C15:0 *anteiso*;  C16:0 *iso*; | ND | ND | C16:0 *iso*; C-17:0 *anteiso* | C15:0 *anteiso*; C16:0; C17:0 *anteiso* | C15:0 *anteiso*; C17:0 *anteiso*; C17:0 *iso* |

Strains: 1, 14.2; 2, ‘*S. nigra*’ 452^T^; 3, *S. albogriseolus* DSM 40003^T^; 4, *S. iakyrus* DSM 40482^T^; 5, ‘*S. cupreus*’ PSKA01^T^; 6, *S. cinnabarinus* DSM 40467^T^; 7, *S.* *davaonensis* DSM 101723^T^; ND Not determined; *Data were taken from Chen et al. (2018), **Data were taken from The Compendium of Actinobacteria (<https://www.dsmz.de>), ***Data were taken from Maiti and Mandal (2021), ^#^Data were taken from Landwehr et al. (2018).

**Table S9** Oligonucleotides used for cloning.

| **Primer name** | **Introduced restriction site** | **Sequence** |
| --- | --- | --- |
| rosA_fwd_NdeI | *Nde*I | 5’-AAACATATGCGGCAGGAACCGACCG-3’ |
| rosA_rev_HindIII | *Hin*dIII | 5’-TTTAAGCTTGTCCGCCGTGGCCCGGCATT-3’ |
| rosB_fwd_NdeI | *Nde*I | 5’-AAACATATGGCTCTCAAGGCTCTCATTCTC-3’ |
| rosB_rev_HindIII | *Hin*dIII | 5’-TTTAAGCTTGCCGAGTTGGGCGTCCTC-3’ |
| rosC_fwd_NdeI | *Nde*I | 5’-AAACATATGGTGAGTGACGGACGCGAGAG-3’ |
| rosC_rev_HindIII | *Hin*dIII | 5’-TTTAAGCTTCACGAGGCCGGACGGGGC-3’ |


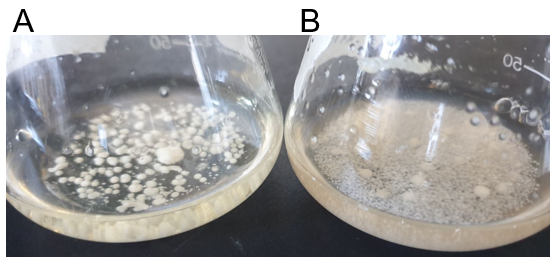


**Figure S1** Growth of isolate *Streptomyces* 14.2 on YS broth. About 50,000 spores were used to inoculate these cultures. The liquid cultures were aerobically grown at 30 °C for 30 h and formation of either large pellets (left flask) or mainly small pellets (right flask) was observed. Roseoflavin formation only started in the right flask containing small pellets.

**
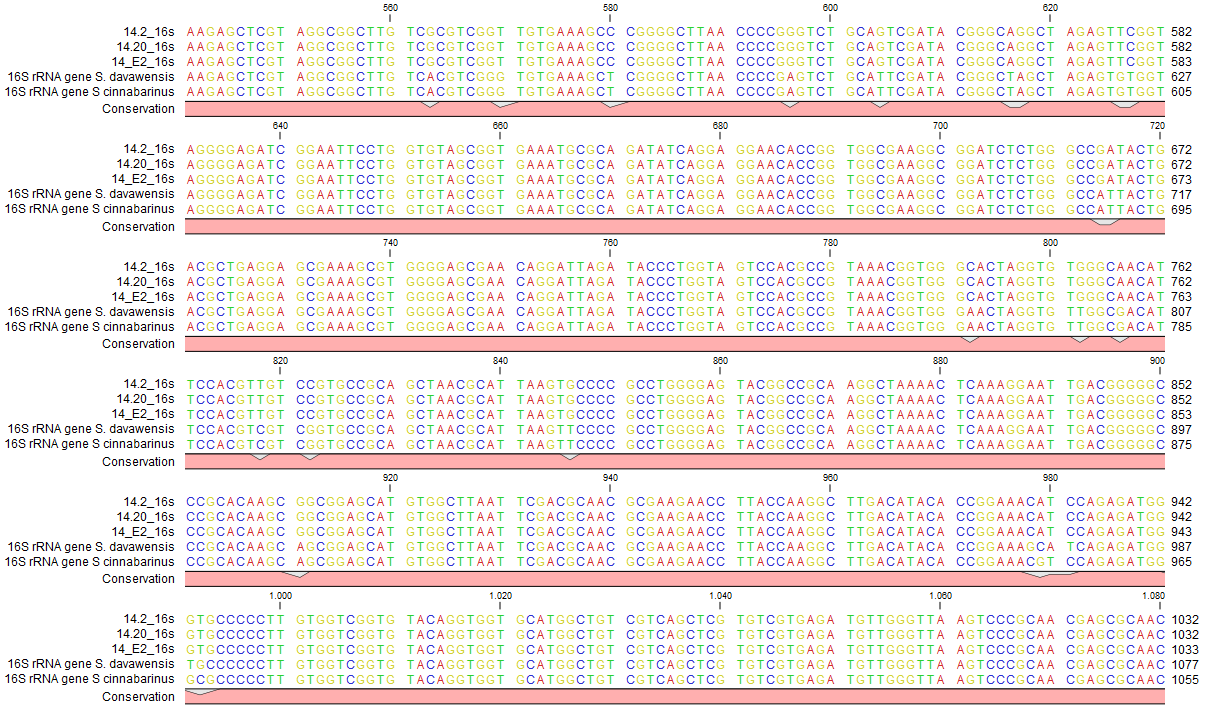
**

**Figure S2** Alignment of the variable regions of 16S rRNA genes from three independent roseoflavin releasing isolates (named 14.2, 14.20 and 14_E2) from Berlin soil samples (s. Fig S3) with the 16S rRNA gene sequences from the roseoflavin producers *Streptomyces davaonensis* and *Streptomyces cinnabarinus*. A break within the thick red line indicates differences between the aligned sequences.


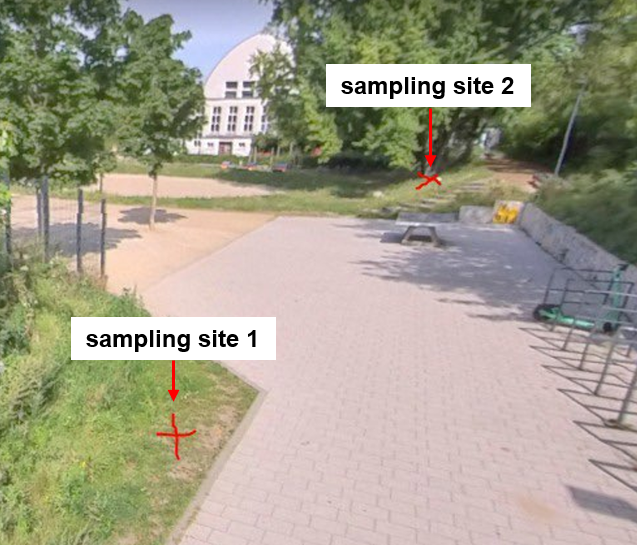


**Figure S3** The sampling sites in “Fritz Schloss Park” in the German capital Berlin (52°31'45.4"N13°21'35.9"E) where *Streptomyces* 14.2 was isolated. Soil samples were taken from two different locations at three different times. The same roseoflavin producing Streptomycete could be isolated as shown by 16S rRNA sequencing (see Fig. S2).


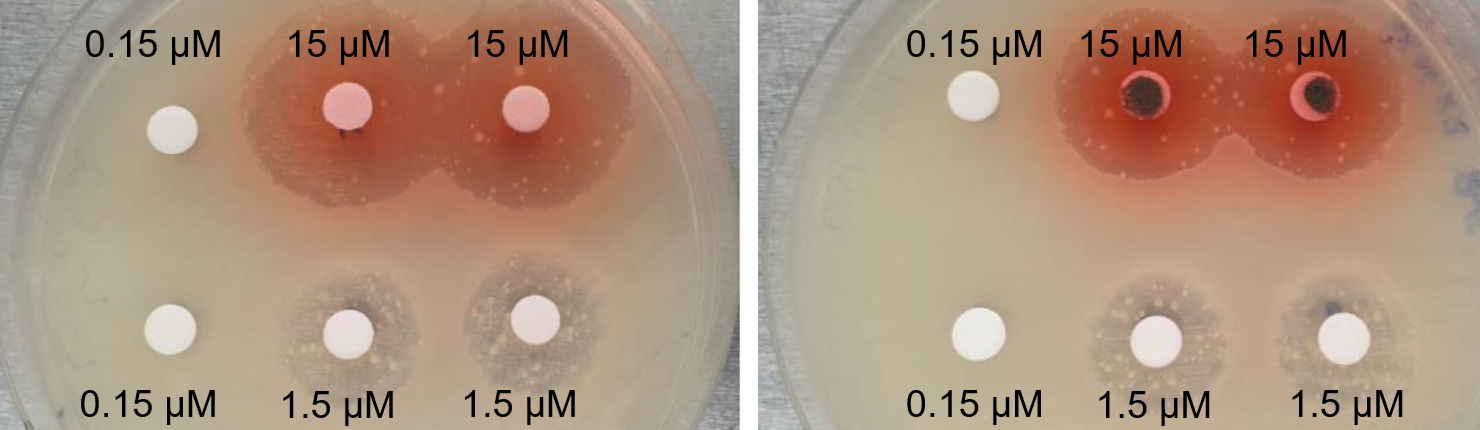


**Figure S4** Roseoflavin susceptibility test using an agar diffusion assay validating the antibiotic activity of the red pigment isolated by preparative high-performance liquid chromatography from a culture supernatant of *Streptomyces* 14.2 (left panel). In the right panel commercially available roseoflavin was tested at indicated concentrations (top rows, 0.15 µM and 15 µM; bottom rows, 0.15 µM and 1.5 µM). *Bacillus subtilis* 168 (DSM23778) was employed as an indicator organism.


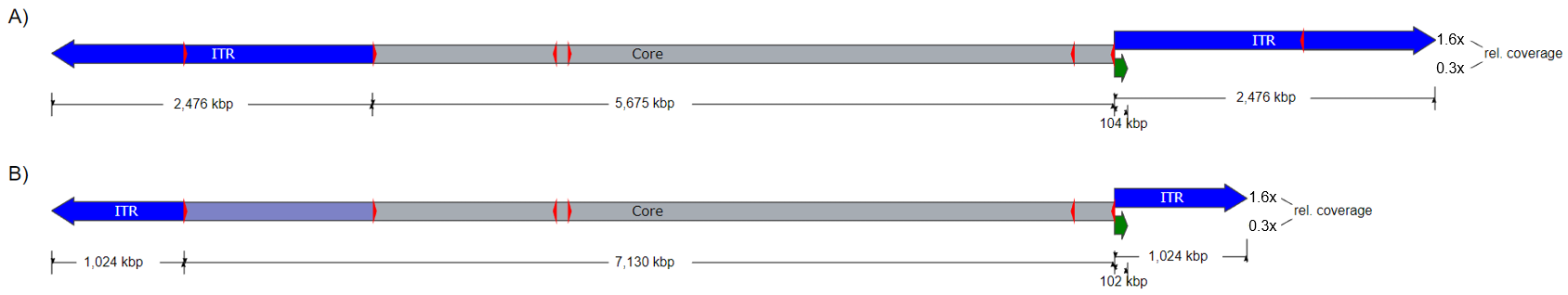


**Figure S5** Schematic representation of the two possible chromosomal arrangements derived from two different genome sequencing projects (A and B). Approach A yielded a linear chromosome with a size of 10.6 Mbp containing a core region of 5.075 Mbp (grey) flanked by two 2.476 Mbp inverted terminal repeats (ITR, blue). Approach B yielded a core region of 7.130 Mbp (grey and faded blue) flanked by two 1.024 Mbp ITRs (blue). In B the core region is larger and the ITR regions are shortened by exactly the sequence (faded blue) that was found in the ITR regions in the first sequencing runs. Both assemblies show an underrepresented third contig (green) which is not part of the core region and is linked to only one side.

**
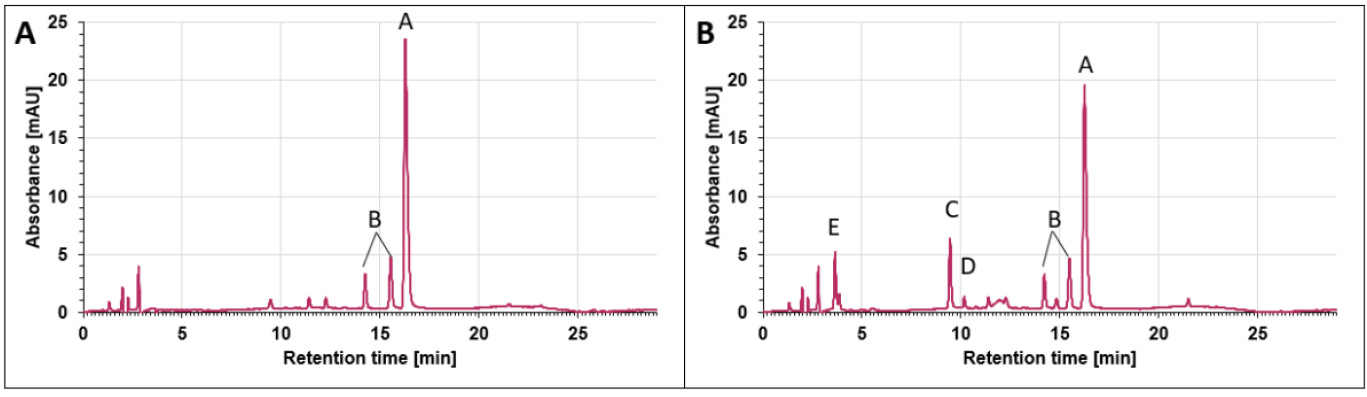
**

**Figure S6** *In vitro* formation of 8-demethyl-8-amino-riboflavin-5’-phosphate (AFP) and the intermediates 8-demethyl-8-formyl-riboflavin-5’-phosphate (OHC-RP) and 8-demethyl-8-carboxyl-riboflavin-5’-phosphate (HOOC-RP) from riboflavin-5´-phosphate (RP or FMN) by RosB from *Streptomyces* 14.2 overproduced in *Escherichia coli* (see also Fig. 1). HPLC-chromatograms show the reaction at 0 hours (panel A) and following incubation for 20 hours (panel B). A cell free extract of a RosB-overproducing recombinant *E. coli* strain was used (5mg total protein) in an enzyme assay containing 1 mM RP at 39°C (Konjik *et al.* 2017). Panel A shows the educt of the reaction (RP = peak A) in addition to two RP isomers (peaks B). Panel B shows the formed product AFP (peak C) and the characteristic reaction intermediates (D = HOC-RP and E= HOOC-RP).

**
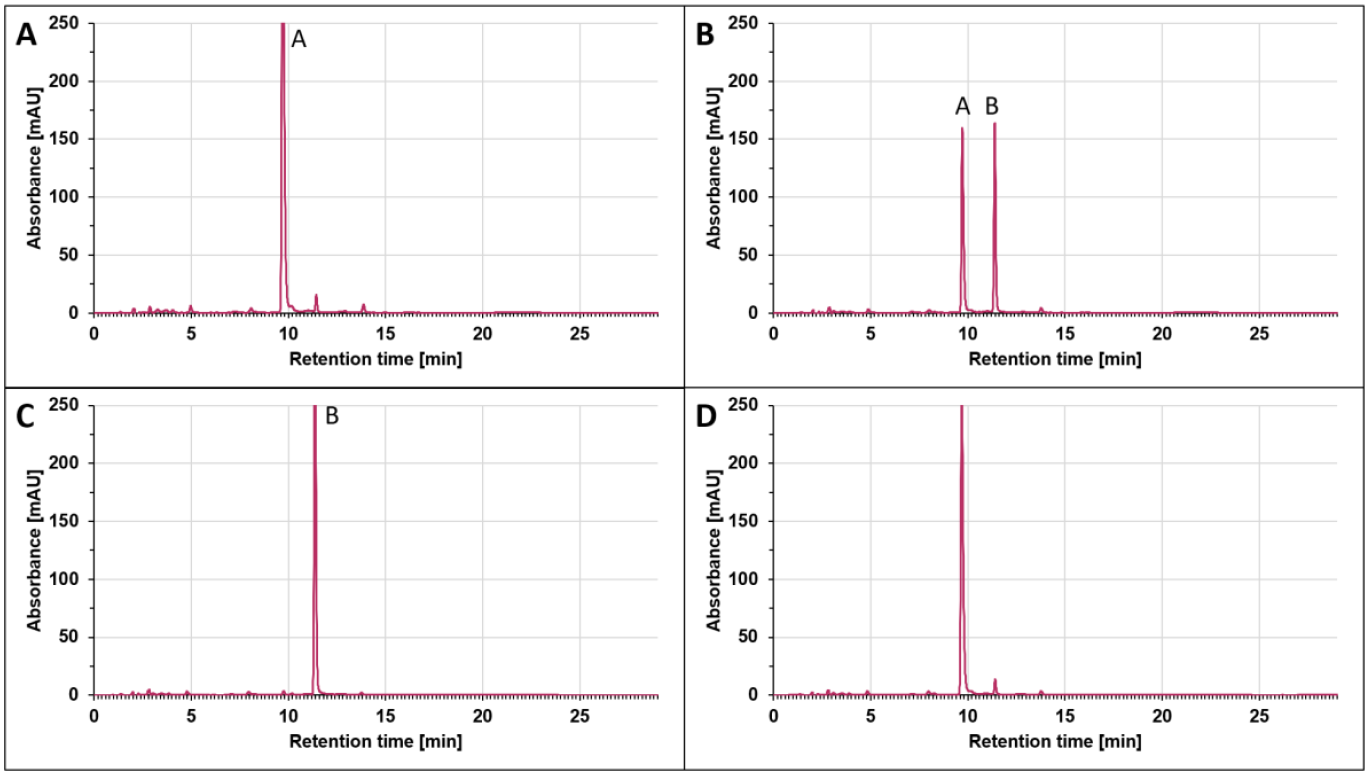
**

**Figure S7** *In vitro* dephosphorylation of 8-demethyl-8-amino-riboflavin-5’-phosphate (AFP) to 8-demethyl-8-amino-riboflavin (AF) by RosC from *Streptomyces* 14.2 overproduced in *Escherichia coli* (see also Fig. 1). The HPLC chromatograms show the reaction at 0 hours (panel A), after 1 hour of incubation (panel B) and after 20 hours (panel C) (37°C). A cell free extract of an *E. coli* strain overexpressing *rosC* from *Streptomyces* 14.2. was used (1 mg of total protein) and tested in an enzyme assay containing 100μM AFP. Panel A shows the educt (AFP = peak A), panel B shows remaining AFP (peak A) and formed AF (peak B). Panel C shows the full depletion of AFP and a single product peak (AF = peak B) after 20 hours. An *E. coli* strain containing the empty expression plasmid (panel D) did not produce AF after 20 h. The presence of small amounts of AF can be explained by the fact that enzymatically prepared AFP contains traces of unreacted AF (Schneider *et al.* 2020).

**
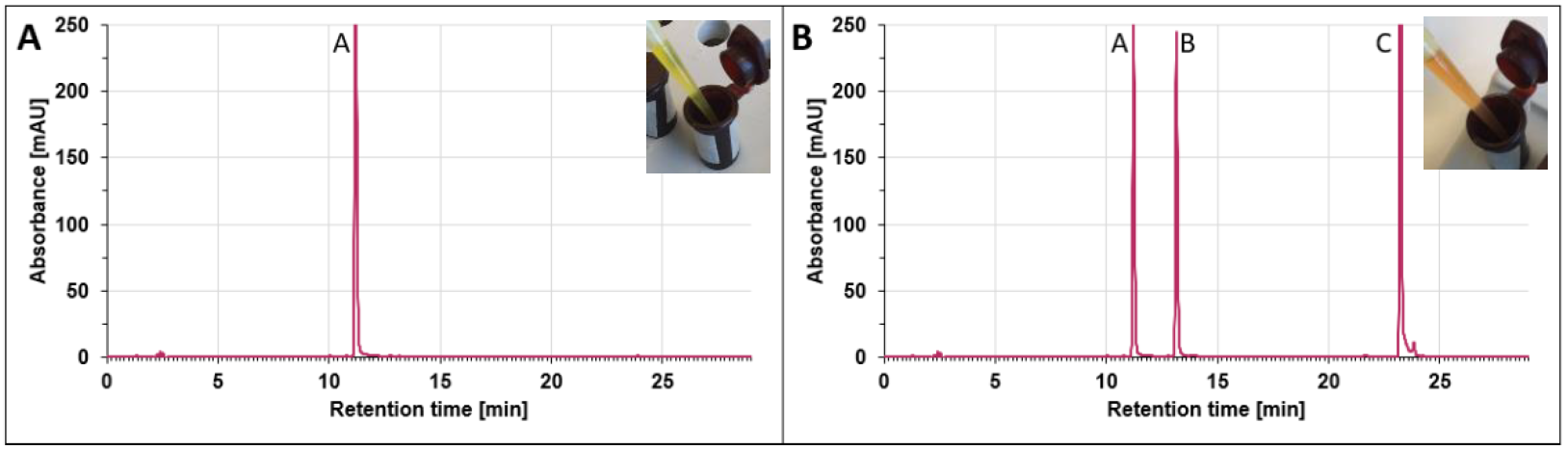
**

**Figure S8** *In vitro* formation of roseoflavin (peak C) and the intermediate 8-demethyl-8-(methylamino)-riboflavin (MAF, peak B) from 8-demethyl-8-aminoriboflavin (AF, peak A) by RosA from *Streptomyces* 14.2 overproduced in *Escherichia coli* (see also Fig. 1). HPLC-chromatograms show the reaction at 0 hours (A) and after 20 hours (B) (37°C). A cell free extract of an *E. coli* strain overexpressing *rosA* from *Streptomyces* 14.2. was used (5mg of total protein) and tested in 50 mM TRIS, 200 μM AF and *S*-adenosyl methionine (Jankowitsch *et al.* 2011). After 20 hours, the reaction was stopped by the addition of trichloroacetic acid. The reaction mixtures, showing the color change from yellow AF to red RoF, are shown in the insets.


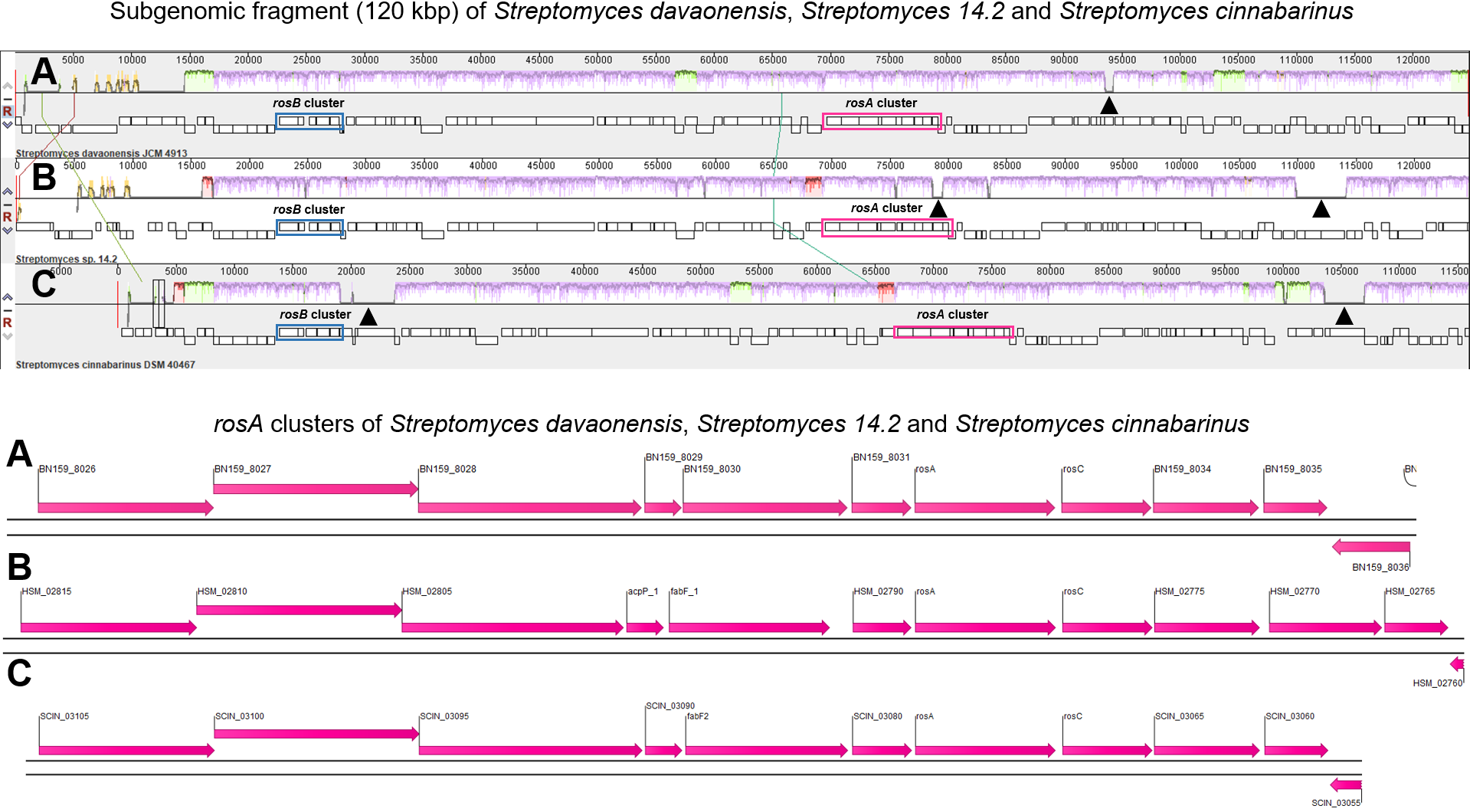


**Figure S9** The roseoflavin biosynthetic genes are found in a 120 kbp subgenomic DNA fragment (top panel) in *Streptomyces davaonensis* (A) (Jankowitsch *et al.* 2012), *Streptomyces* 14.2 (B) and *Streptomyces cinnabarinus* (C) which is similar in the three different roseoflavin producers. The alignment (top panel) was constructed using MAUVE (multiple alignment of conserved genomic sequence with rearrangements) (Rissman *et al.* 2009). Highly similar genes present in all three organisms are marked violet, genes present in *S. davaonensis* and *S. cinnabarinus* only are marked green. Genes present in *Streptomyces* 14.2 and *S. cinnabarinus* are marked red, and genes present in *Streptomyces* 14.2 and *S. davaonensis* are marked yellow. Unique regions are labelled with a black arrowhead. The *rosA* gene clusters are boxed pink in all strains whereas the *rosB* gene clusters are boxed blue. The *rosB* gene clusters contain *rosG*, *ribE2*, *ribX*, *ribY* and *rosB*. The gene *rosG* encodes a putative γ-glutamyltransferase/glutathione hydrolase-like enzyme. Possibly, this enzyme generates glutamate which is the amino group donor in the reaction catalyzed by RosB (**Fig. 1)** (Konjik *et al.* 2017). The gene *ribE2* encodes a riboflavin synthase and is of archaeal origin (Jankowitsch *et al.* 2012). Although many genes are strongly conserved, clear differences in the genetic arrangement are present in the three different bacterial species. The bottom panel shows the *rosA* gene clusters of the three roseoflavin producers. *Streptomyces* 14.2 (B) contains an additional gene with unknown function (HSM_02770) which is not present in the other species.


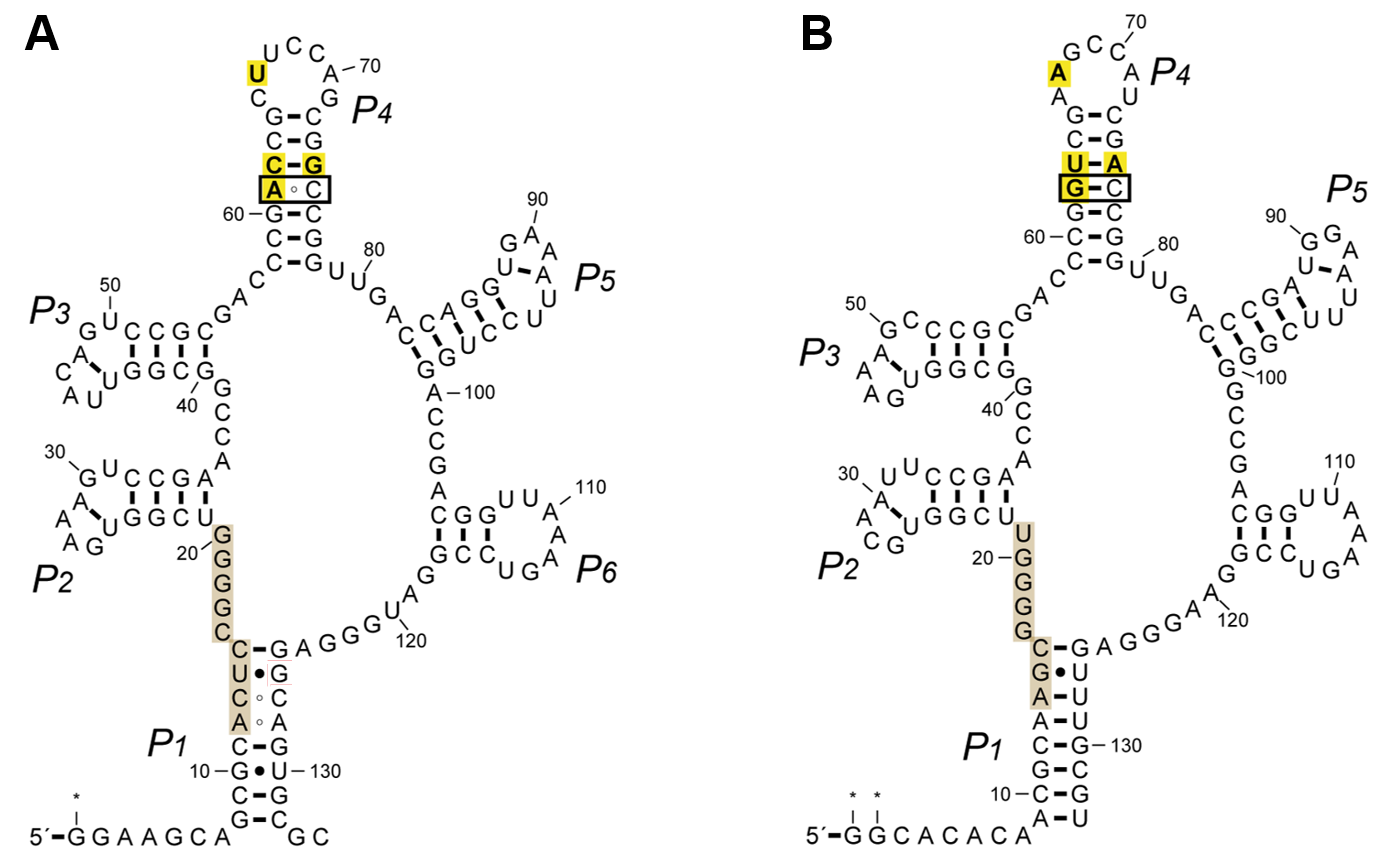


**Figure S10** Aptamer portions (5’ to 3’) of two flavin mononucleotide (FMN) riboswitches, *ribE1* (A) and *ribB9* (B), from *Streptomyces* 14.2. The *ribE1* FMN riboswitch controls expression of the riboflavin biosynthetic genes *ribE1MAB5H*. The *ribB9* FMN riboswitch controls expression of the riboflavin biosynthetic gene *ribB9*. The nucleotides marked brown are responsible for anti-termination/anti-sequestration. Both FMN riboswitches with identical nucleotide sequences are also present in *S.* *davaonensis*. In this bacterium it was shown that the nucleotide pair A61/C75 (A, boxed) is responsible for roseoflavin (RoF) resistance (Pedrolli *et al.* 2012). In contrast, the corresponding nucleotide pair G61/C75 (B, boxed) of the *ribB9* FMN riboswitch is negatively affected by roseoflavin (i.e. roseoflavin mononucleotide, RoFMN) and is responsible for roseoflavin sensitivity.


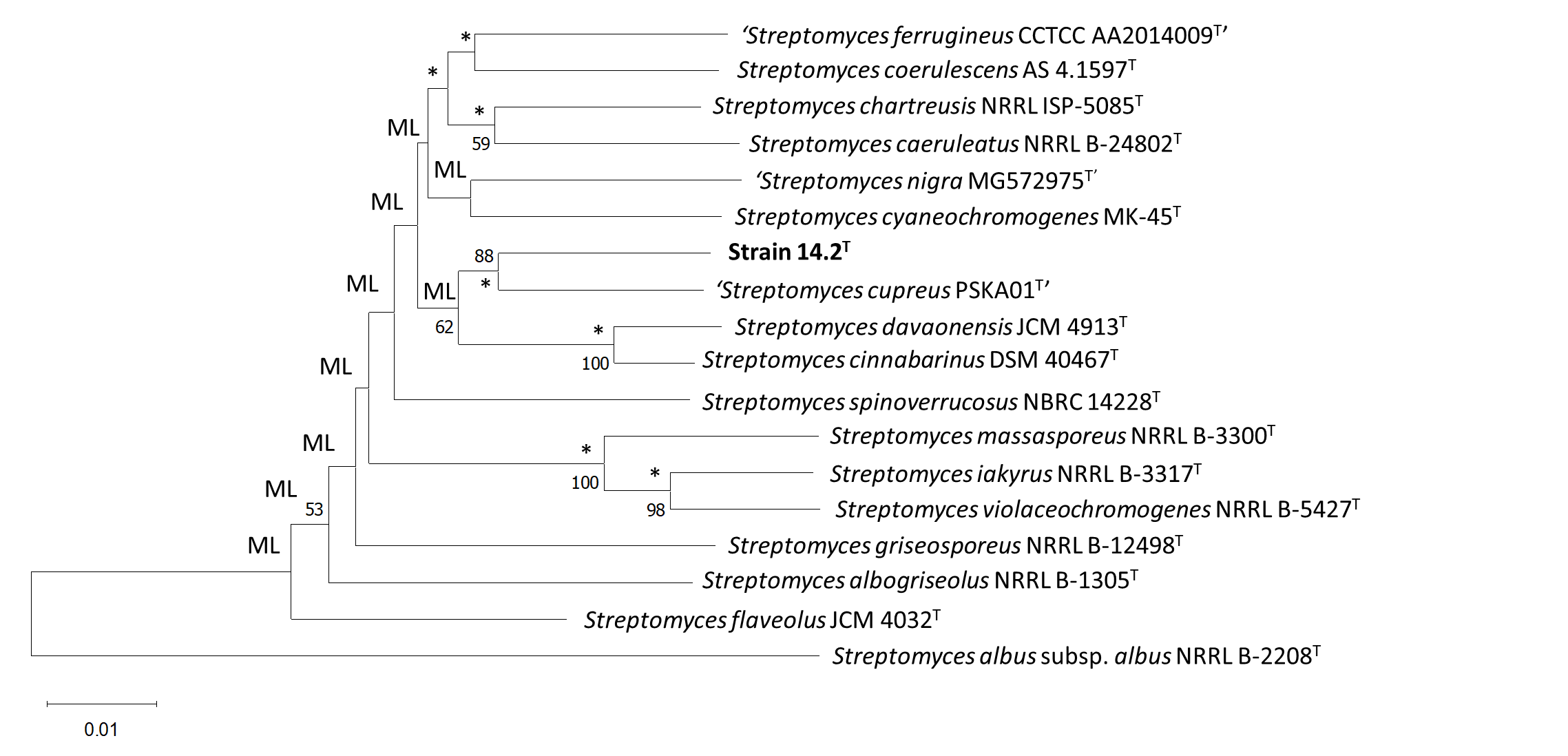


**Figure S11** Neighbour-joining trees based on five-gene concatenated sequences (*atpD*, *gyrB*, *recA*, *rpoB*, and *trpB*). Maximum-likelihood (ML) indicates branches of the tree that were also recovered using the ML method. The asterisk labels branches that were supported by all three tree-making algorithms (neighbor-joining, maximum-likelihood, and maximum parsimony). The scale bars indicate 0.01 nucleotide substitutions per nucleotide position.

**
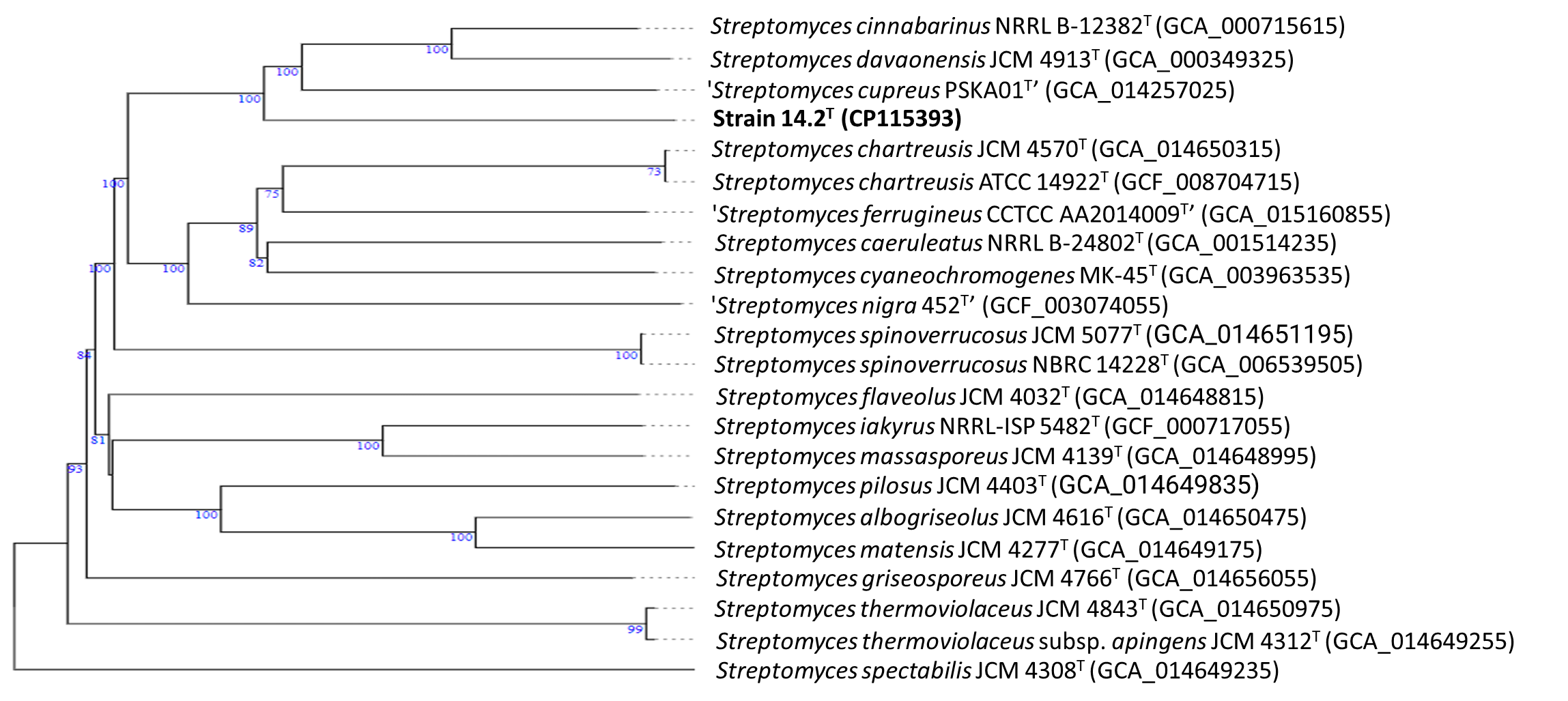
**

**Figure S12** Phylogenomic tree inferred with FastME 2.1.6.1 (Lefort *et al.* 2015) from genome-BLAST distance phylogeny (GBDP) distances calculated from genome sequences. The branch lengths are scaled in terms of GBDP distance formula d5. The numbers above branches are GBDP pseudo-bootstrap support values > 60 % from 100 replications, with an average branch support of 70.9 %.

**
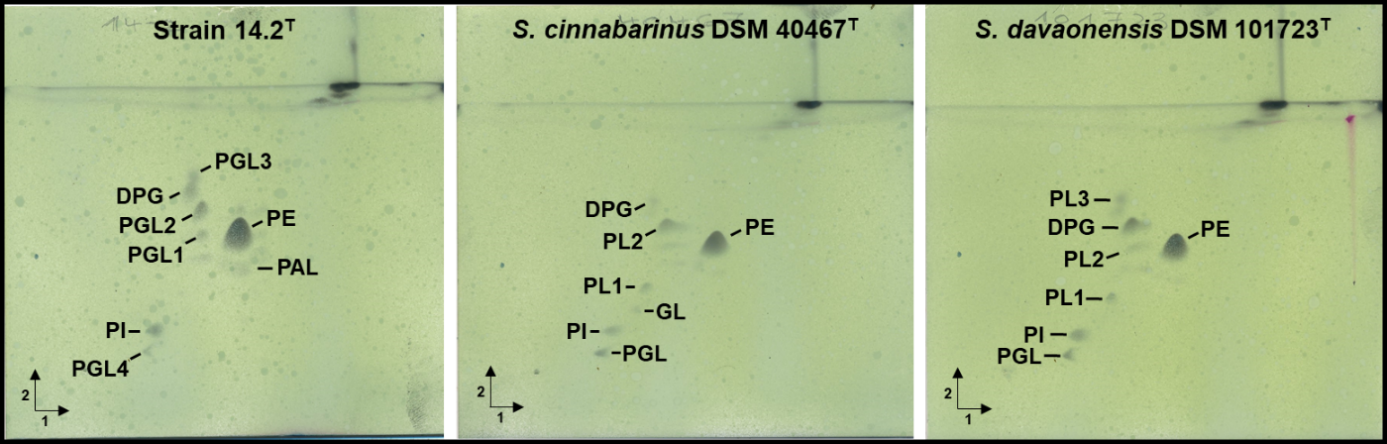
**

**Figure S13** Two-dimensional thin layer chromatography of polar lipids extracted from the roseoflavin producing strains *Streptomyces* 14.2, *Streptomyces cinnabarinus* DSM 40467^T^ and *Streptomyces davaonensis* DSM 101723^T^ stained with molybdophosphoric acid (Sigma P1518). DPG, diphosphatidylglycerol; PG, phosphatidylglycerol; PI, phosphatidylinositol; PAL, phosphoaminolipid; PL1-3, phospholipid; GL, glycolipid; PGL1-4, phosphoglycolipid.


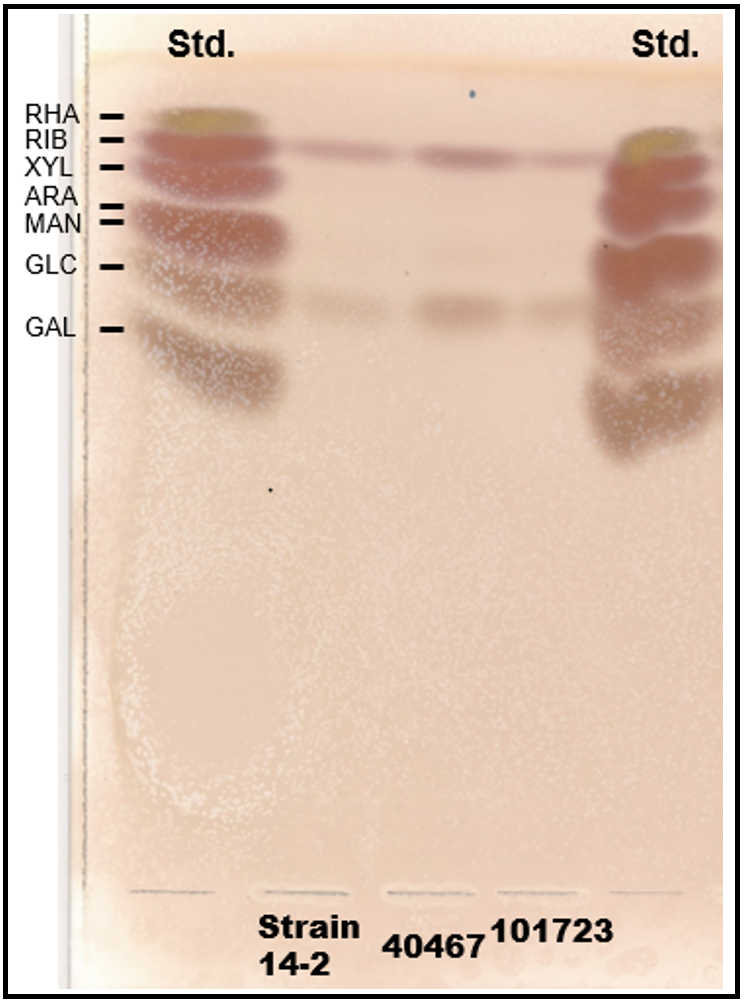


**Figure S14** Thin layer chromatography of whole cell sugars extracted from the roseoflavin producing strains *Streptomyces* 14.2 (novel isolate), *Streptomyces cinnabarinus* DSM 40467^T^ and *Streptomyces davaonensis* DSM 101723^T^. Ribose (RIB) and glucose (GLC) were found to be the predominant sugars present in whole cell hydrolysates.


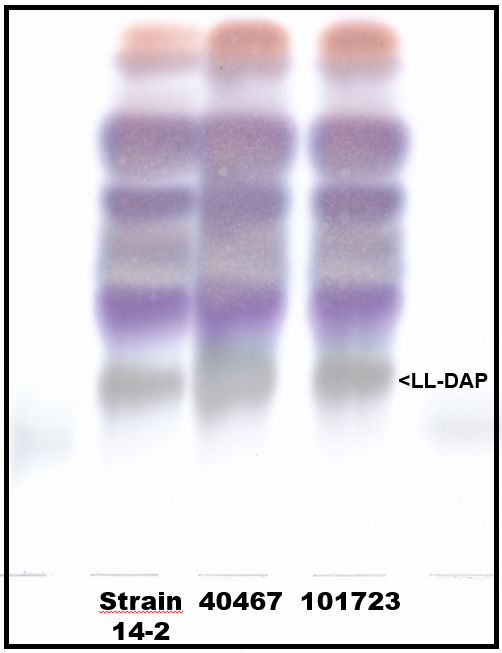


**Figure S15** Separation of the 2,6-diaminopimelic acid (DAP) standard (Sigma) by preparative thin layer chromatography on Cellulose F (Merck) and staining with Ninhydrin reagent. The cell walls of strain *Streptomyces* 14.2^T^ (novel isolate), *Streptomyces cinnabarinus* DSM 40467^T^ and *Streptomyces davaonensis* DSM 101723^T^ contain LL-diaminopimelic acid as the crosslinking diaminoacid.


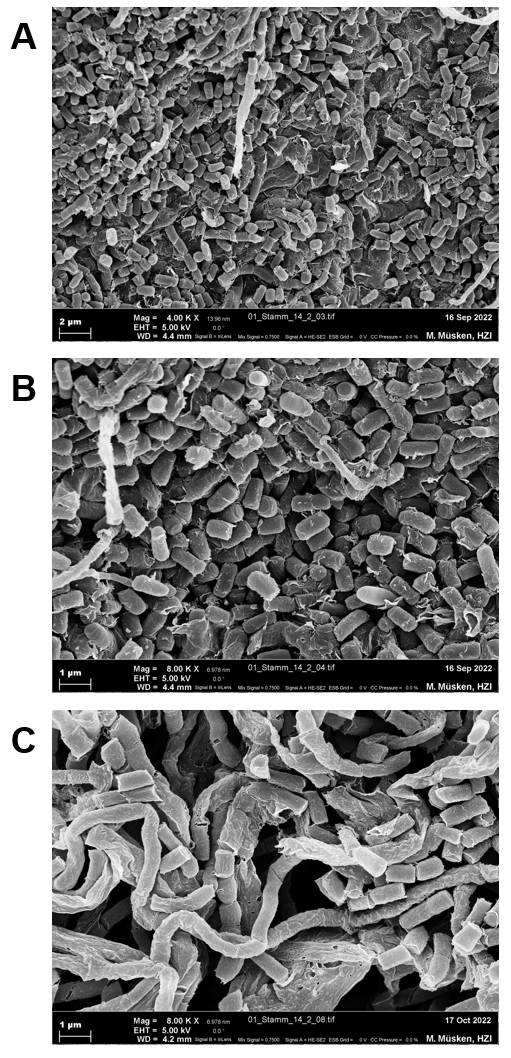


**Figure S16** Electron micrographs of spores of strain 14.2^T^ (new isolate). The size of the cylindrical spores is about 1 µm, the spore surface is slightly rough and the spore chain morphology is “rectus-flexibilis”, spores in straight or flexuous chains.

**References of the supplement**

References

Jankowitsch, F., Kühm, C., Kellner, R., Kalinowski, J., Pelzer, S., Macheroux, P., and Mack, M. (2011) A novel N,N-8-amino-8-demethyl-D-riboflavin Dimethyltransferase (RosA) catalyzing the two terminal steps of roseoflavin biosynthesis in *Streptomyces davawensis*. *The Journal of biological chemistry, doi:* 10.1074/jbc.M111.292300.

Jankowitsch, F., Schwarz, J., Rückert, C., Gust, B., Szczepanowski, R., Blom, J.*, et al.* (2012) Genome sequence of the bacterium *Streptomyces davawensis* JCM 4913 and heterologous production of the unique antibiotic roseoflavin. *Journal of Bacteriology*, **194**: 6818–6827.

Konjik, V., Brünle, S., Demmer, U., Vanselow, A., Sandhoff, R., Ermler, U., and Mack, M. (2017) The crystal structure of RosB: insights into the reaction mechanism of the first member of a family of flavodoxin‐like enzymes. *Angewandte Chemie (International ed. in English)*, **56**: 1146–1151.

Lefort, V., Desper, R., and Gascuel, O. (2015) FastME 2.0: A Comprehensive, Accurate, and Fast Distance-Based Phylogeny Inference Program. *Molecular biology and evolution, doi:* 10.1093/molbev/msv150.

Pedrolli, D.B., Matern, A., Wang, J., Ester, M., Siedler, K., Breaker, R., and Mack, M. (2012) A highly specialized flavin mononucleotide riboswitch responds differently to similar ligands and confers roseoflavin resistance to Streptomyces davawensis. *Nucleic Acids Research*, **40**: 8662–8673.

Rissman, A.I., Mau, B., Biehl, B.S., Darling, A.E., Glasner, J.D., and Perna, N.T. (2009) Reordering contigs of draft genomes using the Mauve aligner. *Bioinformatics (Oxford, England), doi:* 10.1093/bioinformatics/btp356.

Schneider, C., Konjik, V., Kißling, L., and Mack, M. (2020) The novel phosphatase RosC catalyzes the last unknown step of roseoflavin biosynthesis in *Streptomyces davaonensis*. *Molecular Microbiology*.
